# Interpretable convolutional neural networks for sequence-based classification and discovery of plastic-degrading enzymes

**DOI:** 10.1128/aem.01586-25

**Published:** 2026-04-30

**Authors:** Woo-Haeng Lee, Louis Dumontet, KyungMin Jung, Hyun Lee, Gobinda Thapa, Tae-Jin Oh, Mingon Kang

**Affiliations:** 1 Department of Life Science and Biochemical Engineering, SunMoon University 35022 , Asan, Republic of Korea; 2 Department of Computer Science at the University of Nevada, Las Vegas 14722, Las Vegas, Nevada, USA; 3 Department of Computer Science and Engineering, SunMoon University 35022 , Asan, Republic of Korea; 4 Genome-based BioIT Convergence Institute, Asan, Republic of Korea; 5 Department of Pharmaceutical Engineering and Biotechnology, SunMoon University 35022 , Asan, Republic of Korea; Kyoto University, Kyoto, Japan

**Keywords:** plastics, plastic-degrading enzymes (PDEs), hydrolases, classification

## Abstract

The rapid accumulation of plastic waste has emerged as a critical environmental threat, driving the need for scalable and effective biodegradation solutions. Major plastic types, including biopolyesters, aliphatic polyesters, and aromatic polyesters, are widely used in industrial and consumer products and exhibit distinct chemical structures and degradability profiles, posing challenges for systematic enzyme classification. Hydrolytic plastic-degrading enzymes (PDEs) offer a promising solution, yet their functional classification remains limited by insufficient annotations and enzymatic diversity. In this study, we present an explainable deep learning framework, plastic-degrading enzyme prediction via interpretable CNN (PEPIC), to classify enzymes across nine plastic substrate types directly from protein sequences. Using a curated data set of experimentally validated plastic-degrading enzymes (181 sequences) and an expanded homologous data set generated via sequence similarity search (~5,900 sequences), we benchmarked PEPIC against state-of-the-art approaches and evaluated both predictive performance and interpretability. PEPIC demonstrated statistically significant improvements in F1-score compared to state-of-the-art methods. PEPIC calculated contribution scores at the amino acid level, indicating how individual residues influence the predictions. The model interpretation revealed that regions with high contribution scores aligned with key catalytic residues. Homology-based structural modeling demonstrated that residues with high contribution scores mapped to known catalytic and substrate-binding regions of plastic-degrading enzymes and reflected structural differences across plastic classes, supporting the biological relevance of PEPIC’s predictions. PEPIC identified an uncurated enzyme as a potential PET-degrading candidate. This work provides an interpretable framework for plastic-degrading enzyme discovery, which can accelerate biotechnological solutions for plastic waste management and support data-driven strategies toward sustainable environmental remediation.

**IMPORTANCE:**

We propose an explainable deep learning-based approach, named PEPIC, that can effectively classify enzymes across nine plastic substrate categories relevant to hydrolytic PDE activity and provide trustworthy predictions by identifying active and binding sites that align with prior biological knowledge. PEPIC is the first study that demonstrated the high potential of deep learning-based approaches for plastic-degrading enzymes prediction using large data sets. PEPIC not only significantly improved predictive performance compared to the current state-of-the-art models but also provided the trustworthiness of the prediction. PEPIC was thoroughly assessed by intensive and comprehensive experimental settings, and PEPIC enhances the model interpretation for trustworthy predictions and potential new biological knowledge discovery. This work offers scientific advances in accelerating the discovery of plastic-degrading enzymes, contributing to sustainable plastic waste management using a novel AI technique.

## INTRODUCTION

Global plastic production has increased from about 2 million tons in 1950 to 460 million tons in 2019 (1), and nearly 79% of all plastics ever produced have accumulated in landfills or natural ecosystems (2). Polyethylene terephthalate (PET) alone constitutes approximately 10%–12% of total plastic waste and can persist for decades to centuries in the environment (3, 4). Conventional mechanical and chemical recycling approaches have proven insufficient to mitigate this accumulation, often being energy-intensive and economically unsustainable. Consequently, biological degradation has emerged as a promising, eco-friendly alternative for reducing persistent plastic waste.

Plastic-degrading enzymes (PDEs) have emerged as a promising biological solution for the eco-friendly breakdown of synthetic polymers, which attracts significant attention to their structural, catalytic, and ecological diversity (5–7). PDEs, often referred to as plastic hydrolases or depolymerases, catalyze the hydrolysis of synthetic polymers into monomers or oligomers, and they facilitate recycling and upcycling processes. Based on their reaction mechanisms, PDEs are broadly categorized into hydrolases and oxidoreductases. Plastic-degrading hydrolases are typically characterized by a conserved α/β-hydrolase fold core and a catalytic triad and directly cleave ester bonds within polymer backbones (8, 9). On the other hand, oxidoreductases, including laccase, peroxidase, and dioxygenase, primarily participate in the oxidative pretreatment of plastic polymers, particularly nonpolar polymers, by modifying polymer surfaces to enhance accessibility for subsequent hydrolytic reactions (6, 10). Since their activity is often indirect and relies on synergistic interaction with hydrolases, oxidoreductases’ mechanistic roles are difficult to characterize, which limits their usage as direct targets in enzyme engineering and biodegradation studies.

Recent structural and biochemical studies have addressed key features that underlie enzymatic plastic degradation, such as the presence of surface-binding domains (SBDs), hydrophobic patches for polymer interaction, and widened active-site clefts that accommodate polymer chains. For instance, the crystal structure of PETase from *Ideonella sakaiensis* revealed a broadened substrate-binding cleft and a unique serine-histidine-aspartate catalytic triad, which facilitates the hydrolysis of PET (10, 11). These structural characterizations are not only limited to PET-degrading enzymes but also have been observed across other classes of plastic-degrading hydrolases. Similarly, polycaprolactone (PCL) hydrolyzing enzymes often exhibit a lipase-like architecture with a flexible lid domain covering active sites. This lid domain regulates substrate access through conformational switching (12). These enzymes typically possess a hydrophobic binding groove that accommodates the aliphatic backbone of PCL, supporting efficient ester bond cleavage. Polylactic acid (PLA)-degrading enzymes also share the α/β-hydrolase fold and possess broad substrate tolerance, typically originating from esterases or lipases (13). Structural analysis of PLAases reveals active sites tailored for the aliphatic polyester backbone, often coupled with thermostable scaffolds that facilitate activity under industrial conditions (14). Such features have been considered to enhance substrate accommodation and catalytic efficiency on polymeric substrates.

Homology-based sequence search has been the primary method to identify candidate enzymes with evolutionary similarity to known plastic hydrolases (15). The rapid development of high-throughput sequencing technologies has facilitated large-scale mining of metagenomic and proteomic databases for enzyme discovery (7, 15–17). However, reliance on sequence similarity alone presents critical limitations in characterizing plastic-degrading enzymes. Functional divergence frequently occurs without significant changes in sequence identity, often hindering the inference of catalytic activity based solely on homology (7, 18, 19). For instance, PETase from *Ideonella sakaiensis* 201-F6 shares only 51% sequence similarity with PET hydrolase from *Thermobifida fusca*, despite having the same function (20). Furthermore, phylogenetic approaches, such as Enzyme Commission (EC), do not always align with actual substrate specificity: enzymes from the same EC category may degrade different plastic types, and those degrading the same plastic may fall into distinct EC classes. These inconsistencies pose challenges for both annotation and experimental prioritization of enzyme candidates.

Recent advancements in machine learning have demonstrated promising capabilities for protein sequence analysis (21). Notable deep learning models for protein sequence analysis include convolutional neural networks (CNNs) and transformers. CNNs effectively capture sequential dependencies of amino acids, which supports generalization across homologous proteins and enables accurate enzyme function (22, 23). Deep learning and machine learning frameworks for enzyme function prediction demonstrate that these networks can extract informative representations from protein sequences (22–24). Transformers capture long-range interactions and structural context, achieving state-of-the-art performance on large protein data sets as well as enzyme annotation tasks (25–31). Explainable deep learning approaches have identified key functional regions in enzyme sequences and neural representations, which increases trustworthiness and can guide protein design and enzyme engineering (31–36). This predictive performance and interpretability could accelerate the discovery of novel plastic-degrading enzymes while reducing experimental costs.

The current state-of-the-art framework, plastic enzymatic degradation (PED), identifies plastic-degrading enzymes using XGBoost with transformer-based context-aware enzyme sequence representations (19). Despite its successes, PED exhibits several potential limitations. First, the data sets employed for model training and evaluation were relatively small (only 129 sequences belonging to 11 plastic types), which restricts a comprehensive assessment of the model’s robustness across the diverse spectrum of plastic-degrading enzymes. Second, PED considered only the initial 100 amino acids of enzyme sequences, potentially omitting critical functional regions required for accurate enzyme characterization. Lastly, the approach’s reliance on computationally intensive transformers for sequence encoding further restricts its scalability and accessibility.

**Fig 1:**
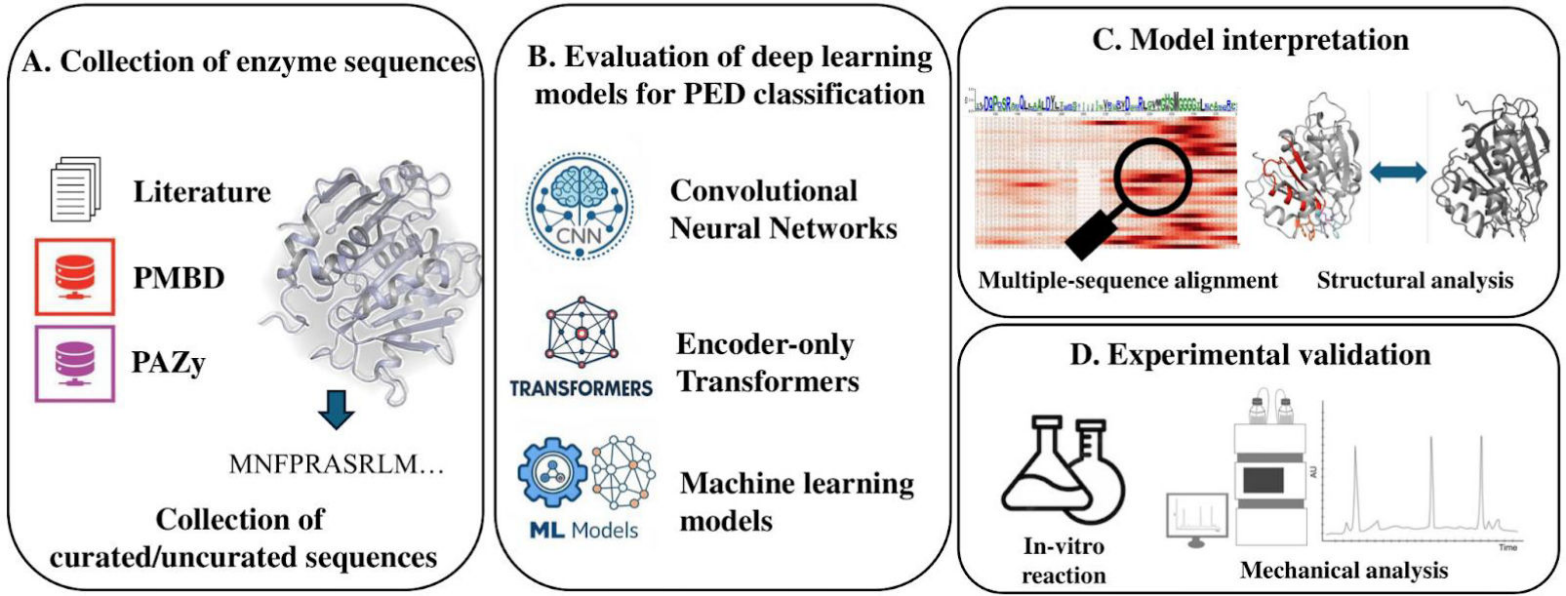
An overview of the study. (**A**) Collection of protein sequences from PMBD, PAZy, and literature having experimental results. (**B**) Deep learning architecture of PEPIC for plastic-degrading enzyme prediction. (**C**) PEPIC provides trustworthy predictions validated by the model’s interpretation by comparing the interpretation with established biological knowledge. (**D**) Biological experiments for the further validation of an uncurated enzyme candidate. PEPIC identified an uncharacterized sequence, and its plastic-degrading activity was confirmed through biological assays.

This study proposes PEPIC (plastic-degrading enzyme prediction via interpretable CNN), an explainable CNN-based deep learning framework for identifying plastic-degrading enzymes from a limited curated data set (Fig. 1). We first establish a data gathering pipeline to curate high-quality, non-redundant enzyme sequences associated with plastic degradation. Our framework identifies plastic-degrading enzyme sequences and integrates an interpretation module, which highlights the motifs within protein sequences that are most relevant to plastic degradation to provide trustworthy predictions. We benchmarked its predictive performance against transformer-based approaches as well as PED, which is the current state-of-the-art method for plastic-degrading enzyme prediction. PEPIC computes contribution scores of amino acid sequences. We validated that the identified amino acids associated with high contribution scores align with established biologically active/binding sites, ensuring the trustworthiness of the predictions. Our findings demonstrated that PEPIC outperforms existing methods for plastic-degrading enzyme prediction and provides interpretability for identifying sequence features associated with plastic degradation. The contributions of this study are as follows: (i) introducing a data-gathering pipeline to assemble a high-quality data set of plastic-degrading enzymes, (ii) proposing an explainable CNN-based deep learning framework for enzyme classification, (iii) integrating an interpretation module that identifies motifs relevant to plastic degradation, and (iv) evaluating the framework’s predictive performance and demonstrating that it yields biologically meaningful interpretations.

## MATERIALS AND METHODS

In this section, we present the architecture of PEPIC, an explainable deep learning framework that predicts plastic-degrading activity from amino acid sequences. We also describe the interpretation strategy for identifying sequence regions that are most relevant to the model’s predictions, providing trustworthiness in the predictions.

### Architecture of the proposed model

**Fig 2:**
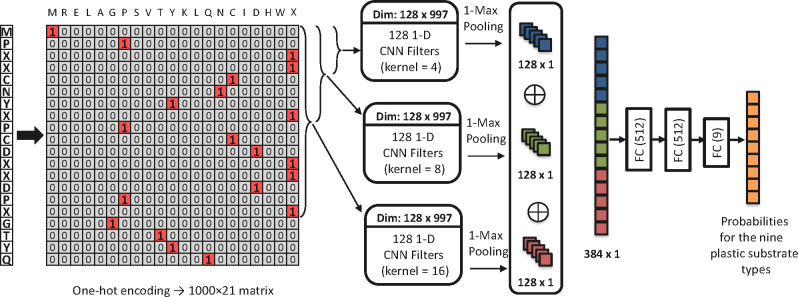
Overview of the PEPIC architecture. The input protein sequence is converted into a 1,000 × 21 one-hot encoded matrix and passed through three parallel 1D convolutional layers with kernel sizes of 4, 8, and 16. After 1-max pooling, the resulting 128-dimensional feature vectors are concatenated, processed through three fully connected layers, and used to predict probabilities for the nine plastic substrate types.

PEPIC analyzes amino acid sequences and predicts enzymes’ plastic-degrading activity. PEPIC employs a convolutional neural network backbone to capture local sequence dependencies, ensure robust generalization across enzyme families, and facilitate interpretability (Fig. 2). PEPIC is designed by adopting the DeepEC architecture for plastic-degrading enzyme prediction (22). PEPIC consists of (i) a protein sequence encoder, (ii) three parallel convolutional layers, (iii) a pooling layer, and (iv) three sequential classification layers. First, the input protein sequences are encoded using an embedding approach. Several embedding schemes were evaluated, including one-hot encoding, BLOSUM62 and ProtVec representations, and one-hot encoding was selected for the final model based on validation performance (Note S2). Second, three convolutional layers are applied in parallel to the encoded sequence using kernel sizes of (4 × *d*), (8 × *d*), and (16 × *d*), where *d* represents the embedding dimension. These kernel sizes were adopted from the DeepEC architecture, which selected them through hyperparameter tuning on large enzyme data sets, and they allow the model to capture sequence patterns at different scales. Smaller kernels detect short functional motifs, while larger kernels capture broader regions related to substrate binding or structural context. Each convolutional layer consists of 128 filters with the ReLU activation, a configuration adopted from DeepEC that provides sufficient representational capacity while avoiding overfitting on curated enzyme data sets. Third, a 1-max pooling operation is applied to each convolutional output to retain the most prominent features. The pooled outputs are then concatenated into a single 384-dimensional feature vector. Finally, this vector is passed through three fully connected layers of sizes 512, 512, and 9, which corresponds directly to the nine plastic-degrading enzyme types considered in this study. The first two layers use ReLU activation, and the final layer applies a sigmoid activation to accommodate the multi-label nature of the task. Model parameters are optimized by minimizing the binary cross-entropy loss between the predicted outputs and the ground truth labels.

### Interpretation strategy

**Fig 3:**
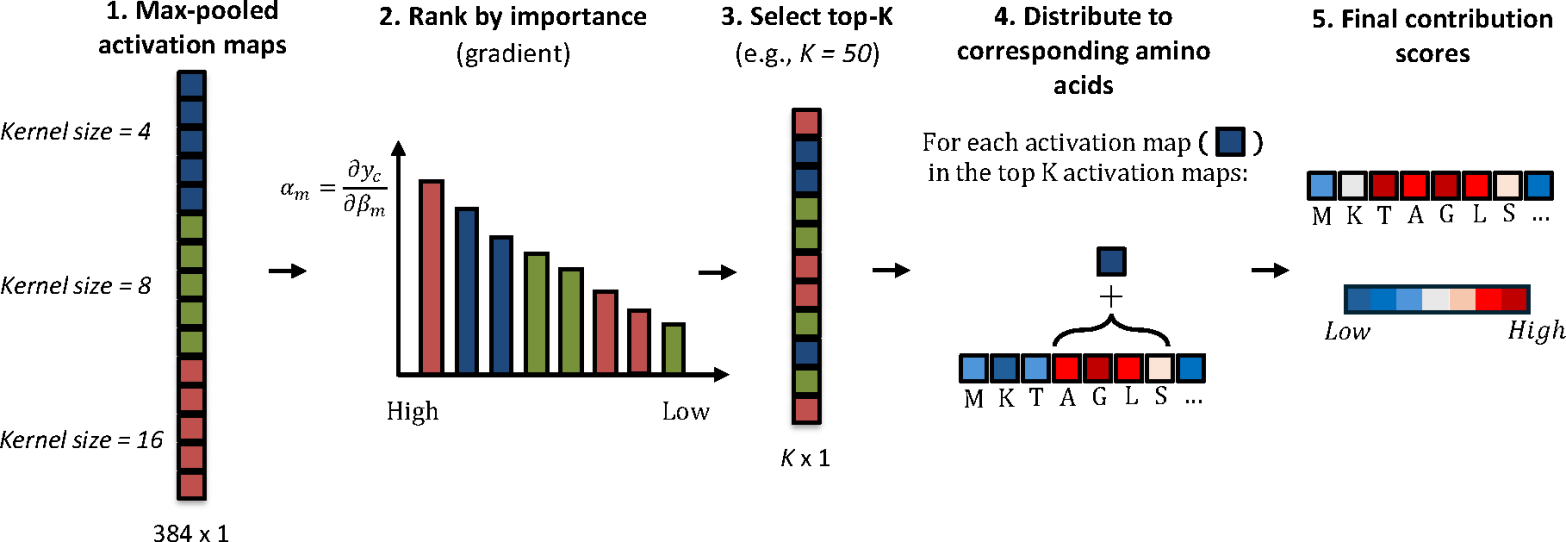
PEPIC interpretation pipeline. Max-pooled activation maps from convolutional filters of multiple kernel sizes are first ranked by gradient-based importance. The top-K most influential activation maps are then selected, and their contributions are distributed back to the corresponding amino acid positions. Aggregating these contributions across all selected maps produces final residue-level contribution scores.

We compute contribution scores for individual amino acids to provide domain-specific evidence, ensuring trustworthiness in the predictions (Fig. 3). The contribution scores identify protein regions, such as conserved motifs or functional domains, by propagating contribution scores from the model’s activation maps. We adapted the explainable deep learning approach developed for CNNs (32). Activation maps are generated by convolutional filters of different sizes (*l* = 4, 8, or 16), each capturing patterns of varying lengths. Each activation map produces a sequence of activation values $\phi_{i}^{k}$, where each value corresponds to a window of *l* consecutive amino acids. Activation maps are ranked based on their contribution to the final prediction (α*_m_*), which is computed as the partial derivative of the prediction score (*y_c_*) with respect to the activation maps’ max-pooled value (β*_m_*), such as:


(1) $$\alpha_{m}=\frac{\partial y_{c}}{\partial \beta_{m}}$$

The top *K* activation maps (e.g., *K* = 50) with the highest scores are selected, and their activation values are summed to compute the contribution score (ζ*_l_*) for each window:


(2) $$\zeta_{l}=\sum_{k=1}^{K}\phi_{i}^{k}$$

Each activation covers multiple amino acids, and its contribution is distributed across individual residues by averaging across all overlapping windows containing amino acid *i*, yielding the contribution score (${\tau_{i}}^{l}$):


(3) $$\tau_{i}^{l}=\frac{1}{l}\sum_{j=1}^{min\left(i,\;j\right)}\zeta_{i-j+1}^{l}$$

Finally, contribution scores from different filter sizes are aggregated to obtain the final amino acid relevance score:


(4) $$\tau_{i}=\tau_{i}^{4}+\tau_{i}^{8}+\tau_{i}^{16}$$

By propagating contribution scores from high-level model activations back to the sequence level, this approach provides an interpretable mapping of functional residues that contribute to a prediction.

## RESULTS

This section first outlines our data gathering pipeline that constructs data sets for model development and evaluation of plastic-degrading enzymes. Second, we assess the predictive performance of PEPIC by comparing it with state-of-the-art benchmark models. Third, we evaluate the trustworthiness of the predictions by aligning PEPIC’s interpretation with established biological knowledge. Finally, we assess the robustness of PEPIC through biological assays on an uncharacterized enzyme candidate predicted by the model.

### Data set collection for model development

We constructed plastic-degrading enzyme data sets for deep learning model development and evaluation. In this study, we focused on hydrolytic enzymes, a well-characterized subclass of plastic-degrading enzymes. We excluded oxidoreductases, due to limited data availability and unclear mechanistic relevance in the context of plastic hydrolysis. First, we collected biologically curated hydrolytic enzyme sequences through a literature review. Then, we generated a model development data set using sequence similarity between UniProt and the biologically curated data set.

#### Curated data sets from literature and PlasticDB

We assembled an experimentally validated data set of plastic-degrading enzymes from peer-reviewed literature and PlasticDB (37). The literature corpus was obtained through a PubMed search (April 2022) using the query: *Plastic* AND (*degrad* OR *depolymer*) AND (bacter* OR fung* OR archaea*). The resulting papers were examined manually, and only enzymes validated through both biochemical assays and analytical characterization were considered. Enzyme candidates, which were supported solely by weight-loss measurements, were excluded since polymer mass loss can result from abiotic processes such as photodegradation, oxidation, or surface erosion (5). The manually curated data set was then cross-referenced with PlasticDB (accessed June 2023). From PlasticDB, we selected sequences annotated as hydrolytic enzymes on the basis of UniProt functional annotations and InterProScan-predicted domains, including alpha/beta-hydrolases, esterases, and cutinases. This approach ensured that all retained sequences exhibited domain-level characteristics associated with hydrolytic activity relevant to plastic degradation. The curated data set covered nine major plastic types, which were divided into three groups according to their chemical structures: (i) biopolyesters such as PHA and PHB; (ii) aliphatic synthetic polyesters such as PBAT, PBS, PBSA, PLA, and PCL; and (iii) aromatic or mixed polyesters such as PET and PU. Representative polymer structures are summarized in Table 1. The resulting curated data set comprised 181 non-redundant sequences, categorized by polymer type as follows: PHA (50, 27.6%), PHB (46, 25.4%), PBAT (16, 8.8%), PBS (11, 6.1%), PBSA (16, 8.8%), PLA (26, 14.4%), PCL (40, 22.1%), PET (75, 41.4%), and PU (14, 7.7%). This data set was used to evaluate the predictive performance of the models.

**TABLE 1:** Summary of plastic types, chemical classes, and representative hydrolytic enzymes covered in the curated data set

Plastic type (acronym)	Full name	Chemical class	Example hydrolytic enzymes
PHA	Polyhydroxyalkanoate	Biopolyester	PHA depolymerase
PHB	Poly(3-hydroxybutyrate)	Biopolyester	PHB depolymerase
PBAT	Poly(butylene adipate-co-terephthalate)	Aliphatic–aromatic copolyester	Cutinase-like hydrolases
PBS	Polybutylene succinate	Aliphatic polyester	PBS hydrolase
PBSA	Poly(butylene succinate-co-adipate)	Aliphatic polyester	Lipase-like hydrolases
PLA	Polylactic acid	Aliphatic polyester	PLA depolymerase
PCL	Polycaprolactone	Aliphatic polyester	PCL hydrolase
PET	Poly(ethylene terephthalate)	Aromatic polyester	PETase, cutinase variants
PU	Polyurethane	Aromatic/aliphatic urethane polymer	Polyurethane esterase

#### Model development data set

We constructed a large model-development data set by expanding the experimentally validated enzyme set using sequence similarity. Potential plastic-degrading enzymes in UniProt were identified through BLASTp (38) searches using the curated enzymes as queries, applying filters of bit-score ≥ 100 and sequence identity ≥ 85%. Sequences with substantial length discrepancies relative to their query enzymes were removed to maintain functional and structural comparability and to minimize contamination from unrelated domains. All retrieved sequences were subsequently clustered using CD-HIT at a 90% sequence identity threshold (39), following established enzyme curation practices (15, 19). The resulting model-development data set contained approximately 5,927 enzyme sequences, categorized by polymer type as follows: PHA (2,657, 44.8%), PHB (1,804, 30.4%), PBAT (253, 4.3%), PBS (75, 1.3%), PBSA (469, 7.9%), PLA (1,195, 20.1%), PCL (1,367, 23.1%), PET (1,473, 24.8%), and PU (313, 5.3%). These values highlight a data set that is enriched in enzymes for PHA, PHB, and PET but remains taxonomically diverse, making it suitable for cross-validation and model training.

We constructed a negative data set of non-plastic-degrading enzymes belonging to the broader α/β-hydrolase superfamily, which includes enzymes that are structurally similar but not associated with plastic degradation. We obtained the negative data set from the Lipase Engineering Database (LED) (40), ensuring coverage of carboxylesterases, lipases, and other related enzymes. To prevent overlapping with the positive data set, we removed all sequences that exhibited above 80% identity similarity with either the biologically curated or model development data sets. Finally, the negative data set comprised 30,350 enzyme sequences. The negative data set was used for computing false discovery rate (FDR).

### Predictive performance comparison using cross-validation and biologically curated data

**Fig 4:**
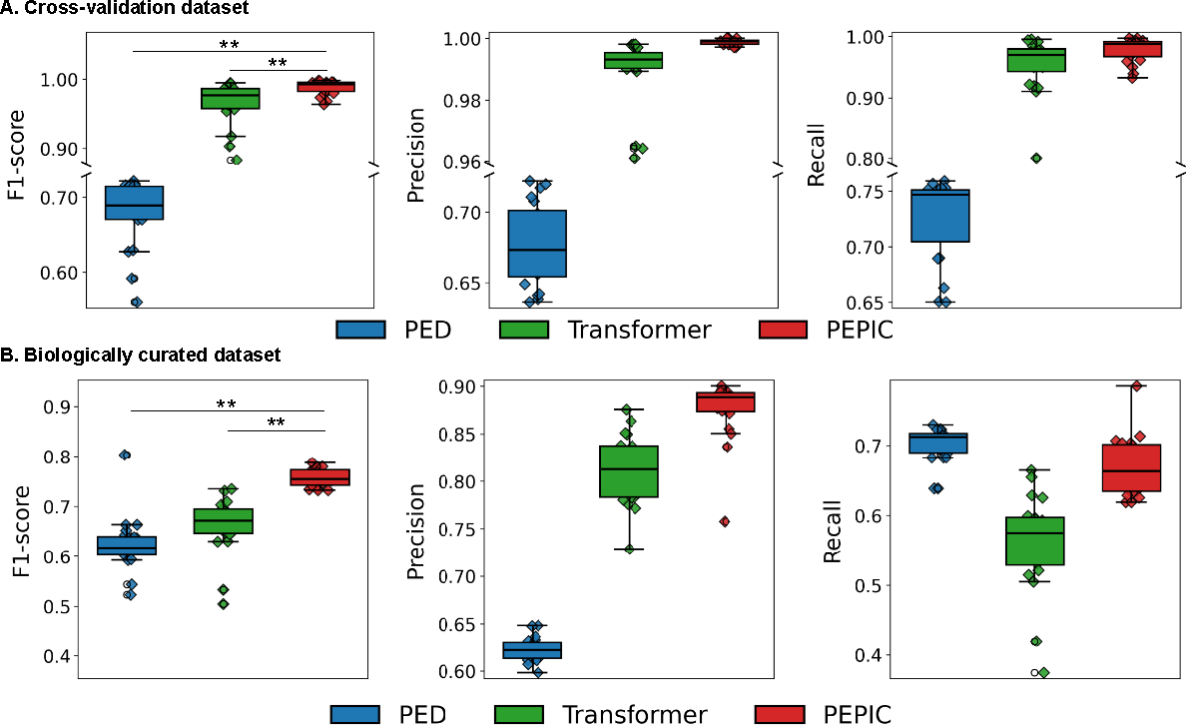
Predictive performance of PED, transformer, and CNN (**A**) on the cross-validation data set and (**B**) biologically curated data set. ** Indicates statistically significant improvements based on the Wilcoxon signed-rank test (*P* < 0.01).

We evaluated the performance of PEPIC to predict hydrolytic plastic-degrading enzymes by comparing it against transformer-based models and PED (19). We considered an encoder-only transformer (41), which is the typical transformer architecture for protein sequence analysis. The details of the transformer architecture are provided in Note S1. For this benchmark experiment, we split the model development data set into training (80%), validation (10%), and test (10%), with stratified sampling to preserve the class ratios. We optimized the models with the training set, and the hyper-parameters were fine-tuned with the validation set. The protein sequence encoder was optimized using the validation set among 13 encoding schemes (42), and the detailed results are presented in the Note S2. The optimal hyper-parameter values of PEPIC were a learning rate of 1e-4, a dropout of 0.1, and protein sequences were encoded with one-hot encoding. PEPIC was trained using the ADAM optimizer. The optimal hyper-parameter values of the transformer model used for comparison were a model dimension of 256, 2 attention heads, 4 encoder layers, a dropout rate of 0.1, and a learning rate of 1e-4. The transformer model was also trained using the ADAM optimizer. For PED, we used the hyper-parameter values reported in the original study (19). Note that its predictive performance has been shown to be relatively non-sensitive to hyper-parameter variations due to the strong inductive bias introduced by the large-scale pre-trained transformer it employs. We assessed the predictive performance of all models on the test data set by computing the micro-averaged F1-score, precision, and recall. We selected the optimal thresholds for the discriminative function that maximize micro-averaged F1 score on the validation set. We also reported the FDRs computed from amino acid sequences in the negative data set. We used the same training, validation, and test data sets for all benchmark models, and the experiment was repeated 20 times for reproducibility. PEPIC significantly outperformed transformer-based models and PED on the cross-validation data set (Fig. 4A). PEPIC achieved an F1-score of 0.989 ± 0.046, precision of 0.999 ± 0.001, and recall of 0.979 ± 0.009, while the transformer model obtained 0.965 ± 0.014, 0.989 ± 0.005, and 0.954 ± 0.021, respectively. PED achieved an F1-score of 0.679 ± 0.021, precision of 0.677 ± 0.014, and recall of 0.726 ± 0.017. This corresponds to an improvement of approximately 2.5% in F1-scores over the transformer model and 44% over PED. All reported ± values correspond to 95% confidence intervals computed across the 20 repeated hold-out experiments. The superiority of PEPIC over the benchmark models was statistically validated using a Wilcoxon signed-rank test, yielding a *P*-value of less than 0.01.

We applied the 20 models optimized on the cross-validation data set to the biologically curated data set to further evaluate their generalization performance (Fig. 4B). On this biologically curated data set, PEPIC achieved an F1-score of 0.757 ± 0.008, precision of 0.875 ± 0.015, and recall of 0.670 ± 0.019. The transformer model obtained an F1-score of 0.661 ± 0.026, precision of 0.811 ± 0.016, and recall of 0.561 ± 0.033, while PED achieved 0.624 ± 0.025, 0.623 ± 0.006, and 0.701 ± 0.012, respectively. These results correspond to an improvement of approximately 15% over the transformer model and 21% over PED in F1-scores. On the negative data set, the FDR values were 0.029 ± 0.006 for PEPIC, 0.044 ± 0.006 for the transformer, and 0.057 ±0.001 for PED.

### Biological validation of PEPIC’s interpretation

**Fig 5:**
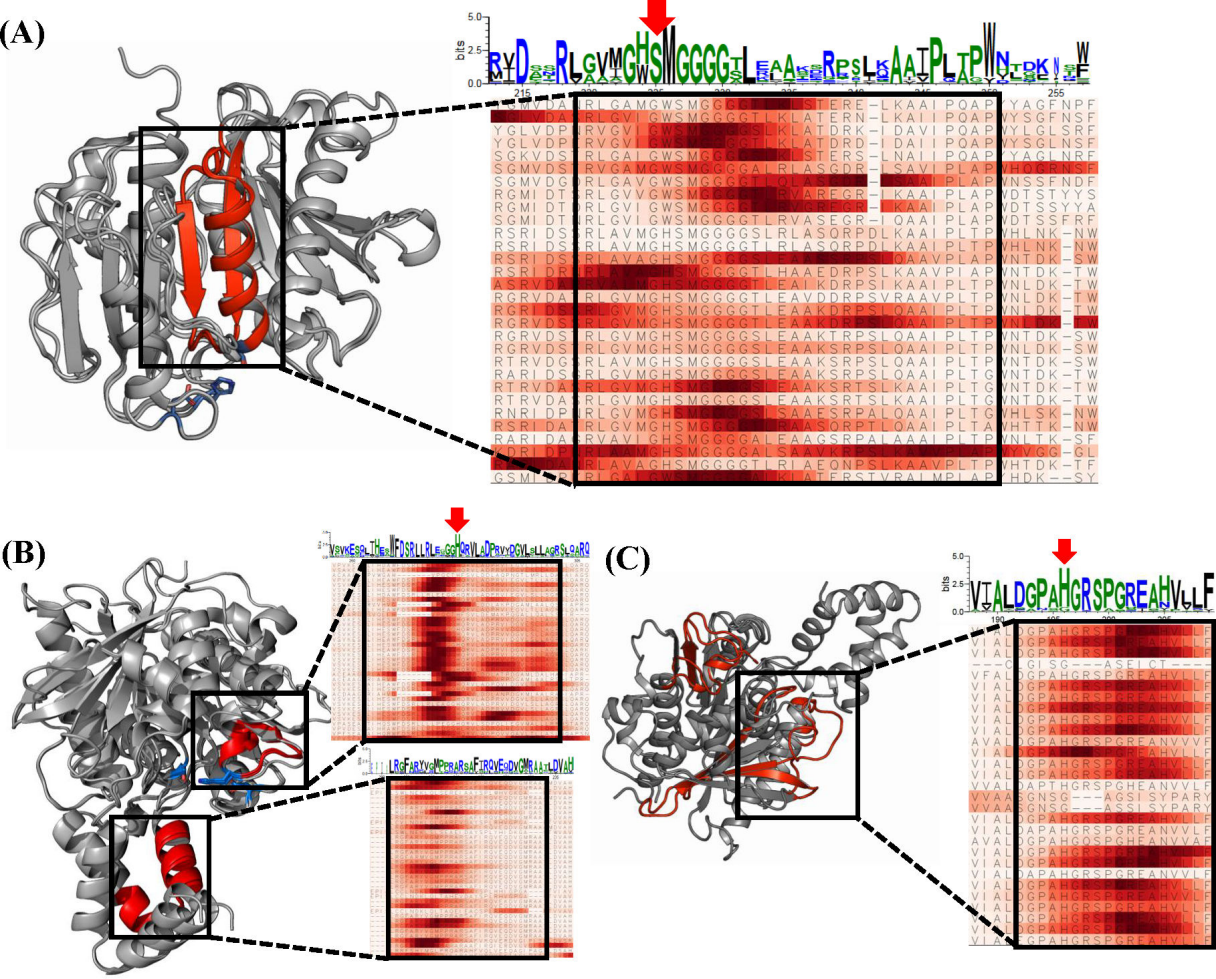
Interpretation results of model-derived importance scores and structural features of key motifs in hydrolytic plastic-degrading enzymes. (**A**) PET-degrading enzyme showing high-contribution regions (red) on the 3D structure and in the sequence heatmap, with catalytic residues (Ser–His–Asp) highlighted in blue and indicated with red arrows in the sequence logo. (**B**) PCL-degrading enzyme with high-scoring regions mapped to substrate-binding loops and lid regions, and the catalytic triad marked in blue. Zoomed-in panels illustrate alignment between conserved histidine motifs (red arrow) and high-contribution regions. (**C**) PLA-degrading enzyme displaying high-contribution regions. Zoomed-in panels illustrate alignment between the conserved histidine motif (red arrow) and high-contribution regions.

We evaluated the biological relevance of PEPIC’s contribution scores to support the trustworthiness of its predictions by comparing high-scoring sequence regions with established functional motifs in plastic-degrading enzymes. For each protein sequence, we computed residue-level contribution scores to identify regions most influential to the model’s prediction. Importantly, these functional motifs, including the Ser–His–Asp catalytic triad and substrate-binding clefts, play essential biochemical roles in hydrolytic plastic degradation: the catalytic triad provides the nucleophilic machinery required for ester-bond cleavage, while the surrounding loops and binding clefts determine polymer accessibility and proper positioning within the active site. By confirming that PEPIC highlights these mechanistically relevant residues rather than arbitrary sequence segments, we ensure that the model’s interpretation aligns with known structural determinants of enzymatic function. For the assessment, we randomly selected PET-, PCL-, and PLA-hydrolyzing enzymes and computed their contribution scores (Fig. 5). We then compared the most influential residues with conserved motifs derived from well-characterized references and mapped them onto homology-modeled structures to verify their localization within catalytically important regions. All the sequences that we used are in Table S1.

For PET-hydrolases, PEPIC showed high contribution scores in subsite I, which includes the nucleophile elbow (Gly-X-Ser-X-Gly motif) (Fig. 5A). The serine residue of the nucleophile elbow plays a critical role in cleaving ester bonds and is a conserved residue among PET-hydrolases. Subsite I is a conserved structural feature composed of an α-helix and β-sheets and is a part of the α/β-hydrolase fold. Specifically, Met226 and Trp/His224 are involved in interactions with the PET polymer, and PEPIC identified them with high contribution scores. Trp260 contributes to substrate-binding affinity through π–π interactions with the aromatic rings of PET (9).

For PCL-hydrolases, PEPIC produced high contribution scores around histidine among the catalytic triads. Specifically, high contribution scores were assigned to the histidine-containing loop (upper box in black) and the helix parts (lower box in black) in Fig. 5B. The histidine-containing loop has high flexibility, which allows flexible adaptation of the substrate binding cleft of the enzyme, thereby increasing substrate specificity (43). This helix part is a variable structure covering the active site observed in lipases (12) and is directly related to substrate specificity, active site accessibility, and reaction mechanism.

For PLA-hydrolases, PEPIC identified several loops and sheets that are distant from the catalytic site (Fig. 5C), as well as histidine-containing loops like PCL-hydrolases. The loops and sheets can be interpreted as structural adaptations to accommodate flexible, aliphatic substrates. Unlike PET-hydrolyzing enzymes, most PCL- or PLA-hydrolyzing enzymes form a pocket-shaped structure to bind aliphatic substrates (13, 14, 44). In particular, the β4–α3 loop is significantly extended compared with that of PET hydrolases, contributing to the formation of a substrate-binding pocket. Similarly, the β3–β4 loop and the histidine-containing loop are known to contribute to shaping the substrate-binding pocket and determining substrate specificity.

### External validation with an uncurated enzyme

**Fig 6:**
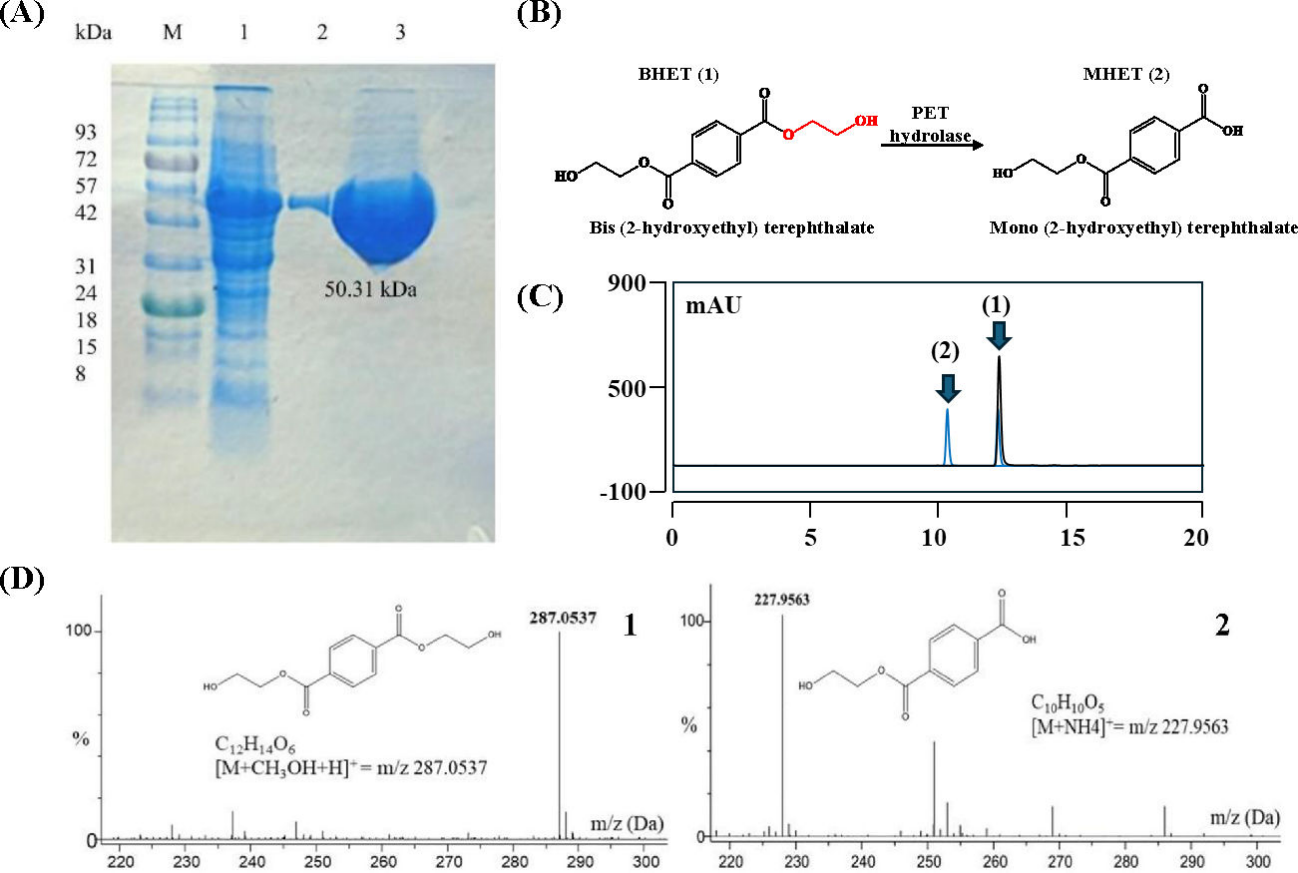
Experimental validation of PET-hydrolytic activity of W2061_PET9. (**A**) SDS-PAGE of recombinant W2061_PET9 expression and purification. Lane M: Molecular weight marker; Lane 1: Whole-cell lysate after IPTG induction; Lane 2: purified W2061_PET9 protein after affinity chromatography; Lane 3: Concentrated W2061_PET9 protein after purification. (**B**) Schematic representation of enzymatic hydrolysis of bis(2-hydroxyl) terephthalate (BHET) into mono(2-hydroxyethyl) terephthalate (MHET). (**C**) HPLC chromatogram of the *in vitro* reaction mixture showing separation of substrate and product. Peak (1) corresponds to BHET, and peak (2) corresponds to MHET. (**D**) Mass spectrometry analysis confirming substrate and product identities. Leaf panel: BHET detected as a methanol adduct ion, Right panel: MHET detected as an ammonium adduct ion.

**Fig 7:**
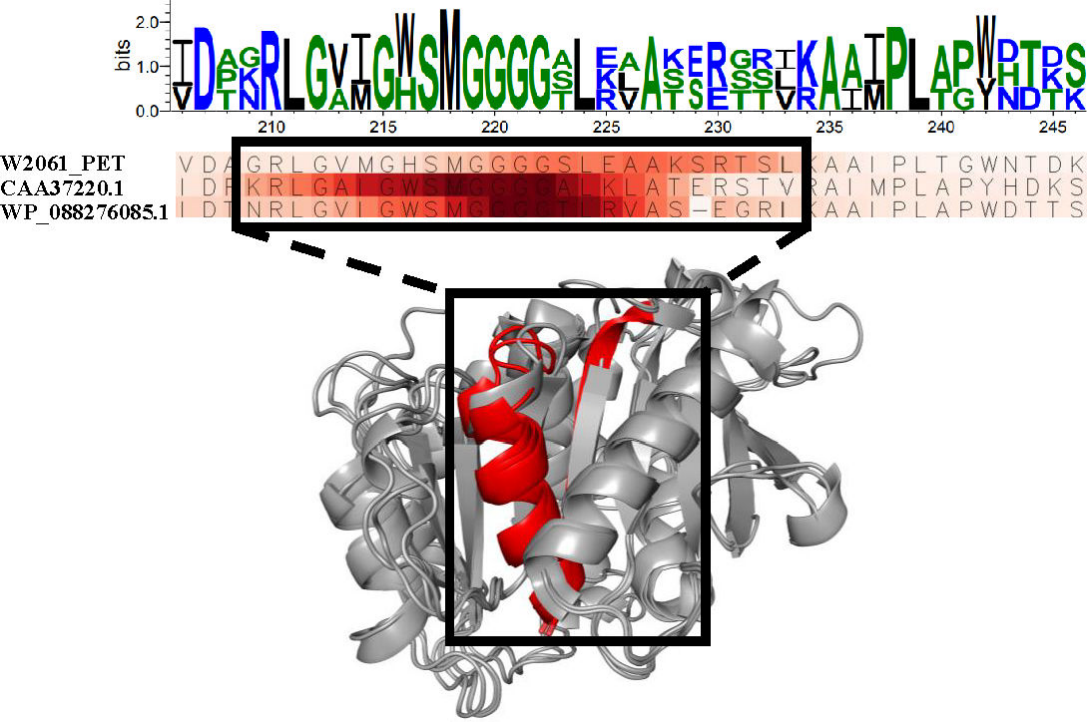
Interpretation results of model-derived importance and structural conservation of a key motif in hydrolytic plastic-degrading enzymes. (Top) Sequence logo generated from a multiple sequence alignment of hydrolytic plastic-degrading enzymes, highlighting conserved residues within the 210–245 amino acid region. (Middle) Heatmap visualization of residue-level importance scores derived from the trained deep learning classification model. Shades of red indicate higher attention weights assigned by the model, with the GXSXG motif showing consistently elevated importance across sequences. This region overlaps with highly conserved residues identified in the sequence alignment. (Bottom) Structural mapping of the high-importance region onto the reference enzyme structure. The red-highlighted segment corresponds to the GXSXG motif and adjacent residues, which are positioned near the substrate-binding pocket. The alignment of model-derived attention with structural and evolutionary conservation suggests a potential role in substrate recognition and catalytic specificity.

We evaluated the robustness and generalization capacity of PEPIC by applying it to a plastic-degrading candidate enzyme that has no prior experimental annotation. To select the best candidate enzymes in this experiment, we performed a local BLAST search on microbial genomes isolated from extreme environments (e.g., psychrophilic- or photosensitizer-associated strains), querying PETase from *Ideonella sakaiensis* (UniProt ID: A0A0K8P6T7). Among them, we found a candidate gene exhibiting low sequence identity (i.e., similarity <40%) and unclear functional annotation but having a conserved domain in multiple-sequence alignment with known PET-hydrolases. We investigated both its enzymatic activity and the interpretability of the model’s prediction to determine the model’s reliability in identifying novel plastic-degrading enzymes beyond curated data sets. PEPIC predicted a 59.3% probability of PET-hydrolyzing activity for W2061_PET9. For biochemical validation, we employed bis(2-hydroxyethyl) terephthalate (BHET) as the assay substrate (see Note S3 for cloning, expression, purification, and HPLC assay protocols). BHET is not only a double and structurally well-defined PET depolymerization intermediate but also the standard substrate used in the initial functional validation of PET-degrading enzymes. In contrast to PET—which is an insoluble, semi-crystalline polymer whose enzymatic degradation strongly depends on crystallinity, film thickness, surface area, and pretreatment—BHET provides a uniform and reproducible reaction environment (5, 45). This reproducibility is essential for quantifying hydrolytic activity, since BHET allows direct measurement of product formation (MHET, TPA) by HPLC or LC-MS, whereas PET assays often yield highly variable or non-quantitative results. BHET is frequently used as a soluble model substrate for evaluating the activity of PET-degrading enzymes, as it enables reliable and quantitative detection of enzymatic turnover without complications arising from polymer heterogeneity (10, 19). In previous studies, including those by Joo et al. (2018) (11) and Yoshida et al. (2016) (20), both BHET and PET substrates were employed to enable complementary assessment of enzyme activity and degradation efficiency. In this context, BHET facilitates quantitative and comparative evaluation of enzymatic activity, while PET-based assays provide validation under polymer-level conditions. Thus, BHET-based assays are widely used as a practical approach for comparative functional screening of PET-degrading candidates prior to polymer-level validation. In the biological experiment, this enzyme successfully cleaved the ester bond in BHET during *in vitro* assays, producing Mono(2-hydroxyethyl) terephthalate (MHET) as the product (Fig. 6A). HPLC and LC-MS analyses of the reaction mixture showed a substrate peak at around 11 minutes and a product peak corresponding to MHET at 10.5 minutes, with molecular weights of m/z 287.0537 and m/z 227.9563, respectively. The recombinant enzyme exhibited measurable hydrolytic activity toward BHET, supporting its role as a PETase-like enzyme catalyzing the initial step in PET deconstruction (Fig. 6B). Furthermore, the interpretation for this candidate enzyme was consistent with those of known PET-hydrolases. As shown in Fig. 7, high contribution scores were observed in subsite I, including the nucleophile elbow. Although the overall score was slightly lower than that of experimentally validated enzymes, the high contribution in the nucleophile elbow suggests that PEPIC effectively identified functionally relevant features.

## DISCUSSION

In summary, we introduce an explainable CNN-based framework, PEPIC, for substrate-specific classification of hydrolytic plastic-degrading enzymes directly from protein sequences. PEPIC achieves state-of-the-art predictive performance while providing residue-level contribution scores that align with known catalytic and substrate-binding regions, enabling biologically meaningful interpretation. PEPIC outperformed existing machine learning and deep learning approaches, demonstrating strong predictive performance on both cross-validation and curated biological data sets (i.e., cross-validation F1-score = 0.989 ± .098, curated data set F1-score = 0.757 ± .018, *P*-value < 0.01 Wilcoxon signed-rank test). Beyond benchmarking, we demonstrate practical utility by experimentally validating a previously uncharacterized PET-degrading enzyme predicted by the model. Together, these results establish PEPIC as an accurate and trustworthy tool for enzyme discovery, offering a scalable computational approach to accelerate the identification of plastic-degrading biocatalysts and support sustainable plastic biodegradation efforts.

However, this study may have potential limitations to be discussed. First, the high predictive performance on the cross-validation may be produced due to the high sequence similarity in the data sets. Nevertheless, PEPIC maintained superior performance on the biologically curated data set that presents more sequence diversity and less redundancy, which demonstrates its robustness. In contrast, transformers exhibited intermediate performance across both data sets. Transformers typically require larger data sets to fully leverage their capacity for modeling long-range dependencies, and the performance is expected to improve with access to broader and more diverse training data. Overall, PEPIC is highly effective for hydrolytic plastic-degrading enzyme prediction in settings with limited curated data.

The current framework is constrained by the availability and composition of experimentally validated plastic-degrading enzyme data sets, which largely reflect the current state of available experimental evidence. Although the curated data set was assembled with careful manual verification to ensure high confidence in functional annotation, the overall number of experimentally characterized plastic-degrading enzymes remains limited relative to the diversity of plastic polymers and degradation mechanisms present in natural environments (17). In particular, the predominance of bacterial plastic-degrading enzymes in the curated and model-development data sets may bias the model toward sequence and structural features characteristic of bacterial hydrolases (20). Consequently, plastic-degrading enzymes from fungi or archaea, which often exhibit distinct secretion strategies, substrate-binding architectures, or adaptations to extreme environments, may not be equally well represented by the current model. This limitation may restrict ecological interpretations of plastic degradation processes in environments where non-bacterial organisms play important roles, such as soil, compost, or extreme habitats. Expanding experimentally validated data sets to include broader taxonomic diversity will therefore be essential for improving the generalizability and ecological relevance of future predictive frameworks.

Building on these observations, future efforts should focus on systematically expanding both the taxonomic and functional scope of available data sets. At the sequence level, this can be achieved through large-scale genome and metagenome mining across diverse ecological niches, followed by homology-based filtering and model-guided prioritization of candidate enzymes for experimental validation. At the experimental level, predicted enzymes should be validated using standardized activity assays against representative plastic substrates, together with protein expression and biochemical characterization. In addition to taxonomic expansion, incorporating a broader range of plastic-degrading enzyme classes, including oxidoreductases, as well as additional plastic types that remain underrepresented in current studies, will further extend the scope and applicability of the proposed framework across diverse plastic degradation contexts.

PEPIC occupies a distinct position within machine learning approaches for enzyme prediction. General EC prediction frameworks such as CLEAN (24), DeepEC (22), and DeepECtransformer (26) classify enzymes using hierarchical EC numbering. However, EC numbers do not uniquely define plastic-degrading activity, and PDEs often fall into broad enzymatic categories that obscure substrate specificity. For this reason, EC-based models may produce ambiguous outputs when applied to plastic-degrading enzymes. PED (19) is targeted to plastic-degrading enzymes but analyzes only the truncated sequence and does not provide residue-level interpretability. PEPIC addresses both challenges by modeling full-length protein sequences and generating contribution profiles that highlight catalytic triads, substrate-binding elements, and conserved fold features associated with PET, PCL, and PLA hydrolysis. This interpretability strengthens the biological relevance of predictions and supports PEPIC as a dedicated framework for identifying plastic-degrading enzymes.

Beyond addressing methodological improvements, enhanced classification of plastic-degrading enzymes also carries important practical implications. By enabling reliable assignment of enzymes to specific plastic classes, PEPIC can support targeted screening strategies for bioremediation, allowing researchers to prioritize enzyme candidates suited to particular polymers or environmental contexts. In addition, the interpretability of the framework provides insights into catalytic and substrate-binding features, which may facilitate rational enzyme selection and engineering for the development of biodegradable materials and plastic upcycling processes. Together, these capabilities help bridge sequence-based enzyme discovery and real-world applications in sustainable plastic waste management.

## Data Availability

Protein sequences used for biological assessment are available in online supplementary fileTable S1. The open-source code is available at https://github.com/datax-lab/Plastic. All other relevant data are available from the authors upon request.

## SUPPLEMENTAL MATERIAL

Supplemental material aem.01586-25-s0001.pdf Notes S1 to S3; Table S1.

ASM does not own the copyrights to Supplemental Material that may be linked to, or accessed through, an article. The authors have granted ASM a non-exclusive, world-wide license to publish the Supplemental Material files. Please contact the corresponding author directly for reuse.

## References

[1] OECD. 2022. Global plastics outlook: policy scenarios to 2060. OECD Publishing, Paris.

[2] Geyer R, Jambeck JR, Law KL. 2017. Production, use, and fate of all plastics ever made. Sci Adv 3:e1700782. doi:10.1126/sciadv.1700782 28776036 PMC5517107

[3] Andrady AL. 2011. Microplastics in the marine environment. Mar Pollut Bull 62:1596–1605. doi:10.1016/j.marpolbul.2011.05.030 21742351

[4] Jambeck JR, Geyer R, Wilcox C, Siegler TR, Perryman M, Andrady A, Narayan R, Law KL. 2015. Marine pollution. Plastic waste inputs from land into the ocean. Science 347:768–771. doi:10.1126/science.1260352 25678662

[5] Danso D, Chow J, Streit WR. 2019. Plastics: environmental and biotechnological perspectives on microbial degradation. Appl Environ Microbiol 85:2019, doi:10.1128/AEM.01095-19 PMC6752018 31324632

[6] Wei R, Zimmermann W. 2017. Non-hydrolytic cleavage of polyethylene terephthalate and related polyesters by a cutinase-like enzyme. World J Microbiol Biotechnol 33:152.28674926

[7] Viljakainen VR, Hug LA. 2021. New approaches for the characterization of plastic-associated microbial communities and the discovery of plastic-degrading microorganisms and enzymes. Comput Struct Biotechnol J 19:6191–6200. doi:10.1016/j.csbj.2021.11.023 34900132 PMC8632723

[8] Tacin MV, Costa-Silva TA, de Paula AV, Palomo JM, Santos-Ebinuma V de C. 2021. Microbial lipase: a new approach for a heterogeneous biocatalyst. Prep Biochem Biotechnol 51:749–760. doi:10.1080/10826068.2020.1855442 33315537

[9] Austin HP, Allen MD, Donohoe BS, Rorrer NA, Kearns FL, Silveira RL, Pollard BC, Dominick G, Duman R, El Omari K, Mykhaylyk V, Wagner A, Michener WE, Amore A, Skaf MS, Crowley MF, Thorne AW, Johnson CW, Woodcock HL, McGeehan JE, Beckham GT. 2018. Characterization and engineering of a plastic-degrading aromatic polyesterase. Proc Natl Acad Sci USA 115:E4350–E4357. doi:10.1073/pnas.1718804115 29666242 PMC5948967

[10] Bhandari S, Poudel DK, Marahatha R, Dawadi S, Khadayat K, Phuyal S, Shrestha S, Gaire S, Basnet K, Khadka U, Parajuli N. 2021. Microbial enzymes used in bioremediation. J Chem 2021:1–17. doi:10.1155/2021/8849512

[11] Joo S, Cho IJ, Seo H, Son HF, Sagong H-Y, Shin TJ, Choi SY, Lee SY, Kim K-J. 2018. Structural insight into molecular mechanism of poly(ethylene terephthalate) degradation. Nat Commun 9:382. doi:10.1038/s41467-018-02881-1 29374183 PMC5785972

[12] Li L, Lin X, Bao J, Xia H, Li F. 2022. Two extracellular poly(ε-caprolactone)-degrading enzymes from Pseudomonas hydrolytica sp. DSWY01T: purification, characterization, and gene analysis. Front Bioeng Biotechnol 10:Art. doi:10.3389/fbioe.2022.835847 PMC8971842 35372294

[13] Kitadokoro K, Kakara M, Matsui S, Osokoshi R, Thumarat U, Kawai F, Kamitani S. 2019. Structural insights into the unique polylactate-degrading mechanism of Thermobifida alba cutinase. FEBS J 286:2087–2098. doi:10.1111/febs.14781 30761732

[14] Hajighasemi M, Nocek BP, Tchigvintsev A, Brown G, Flick R, Xu X, Cui H, Hai T, Joachimiak A, Golyshin PN, Savchenko A, Edwards EA, Yakunin AF. 2016. Biochemical and structural insights into enzymatic depolymerization of polylactic acid and other polyesters by microbial carboxylesterases. Biomacromolecules 17:2027–2039. doi:10.1021/acs.biomac.6b00223 27087107 PMC6886529

[15] Danso D, Schmeisser C, Chow J, Zimmermann W, Wei R, Leggewie C, Li X, Hazen T, Streit WR. 2018. New insights into the function and global distribution of polyethylene terephthalate (PET)-degrading bacteria and enzymes in marine and terrestrial metagenomes. Appl Environ Microbiol 84:e02773-17. doi:10.1128/AEM.02773-17 29427431 PMC5881046

[16] Buchholz PCF, Feuerriegel G, Zhang H, Perez-Garcia P, Nover L-L, Chow J, Streit WR, Pleiss J. 2022. Plastics degradation by hydrolytic enzymes: the plastics-active enzymes database-PAZy. Proteins 90:1443–1456. doi:10.1002/prot.26325 35175626

[17] Zrimec J, Kokina M, Jonasson S, Zorrilla F, Zelezniak A. 2021. Plastic-degrading potential across the global microbiome correlates with recent pollution trends. mBio 12:e0215521. doi:10.1128/mBio.02155-21 34700384 PMC8546865

[18] Pearson WR. 2013. An introduction to sequence similarity (“homology”) searching. Curr Protoc Bioinformatics Chapter 3:3. doi:10.1002/0471250953.bi0301s42 PMC3820096 23749753

[19] Jiang R, Shang L, Wang R, Wang D, Wei N. 2023. Machine learning based prediction of enzymatic degradation of plastics using encoded protein sequence and effective feature representation. Environ Sci Technol Lett 10:557–564. doi:10.1021/acs.estlett.3c00293

[20] Yoshida S, Hiraga K, Takehana T, Taniguchi I, Yamaji H, Maeda Y, Toyohara K, Miyamoto K, Kimura Y, Oda K. 2016. A bacterium that degrades and assimilates poly(ethylene terephthalate). Science 351:1196–1199. doi:10.1126/science.aad6359 26965627

[21] Gelman S, Fahlberg SA, Heinzelman P, Romero PA, Gitter A. 2021. Neural networks to learn protein sequence-function relationships from deep mutational scanning data. Proc Natl Acad Sci USA 118:e2104878118. doi:10.1073/pnas.2104878118 34815338 PMC8640744

[22] Ryu JY, Kim HU, Lee SY. 2019. Deep learning enables high-quality and high-throughput prediction of enzyme commission numbers. Proc Natl Acad Sci USA 116:13996–14001. doi:10.1073/pnas.1821905116 31221760 PMC6628820

[23] Sanderson T, Bileschi ML, Belanger D, Colwell LJ. 2023. ProteInfer, deep neural networks for protein functional inference. eLife 12:e80942. doi:10.7554/eLife.80942 36847334 PMC10063232

[24] Yu T, Cui H, Li JC, Luo Y, Jiang G, Zhao H. 2023. Enzyme function prediction using contrastive learning. Science 379:1358–1363. doi:10.1126/science.adf2465 36996195

[25] Jumper J, Evans R, Pritzel A, Green T, Figurnov M, Ronneberger O, Tunyasuvunakool K, Bates R, Žídek A, Potapenko A, et al.. 2021. Highly accurate protein structure prediction with AlphaFold. Nature 596:583–589. doi:10.1038/s41586-021-03819-2 34265844 PMC8371605

[26] Kim GB, Kim JY, Lee JA, Norsigian CJ, Palsson BO, Lee SY. 2023. Functional annotation of enzyme-encoding genes using deep learning with transformer layers. Nat Commun 14:7370. doi:10.1038/s41467-023-43216-z 37963869 PMC10645960

[27] Lin Z, Akin H, Rao R, Hie B, Zhu Z, Lu W, Smetanin N, Verkuil R, Kabeli O, Shmueli Y, Dos Santos Costa A, Fazel-Zarandi M, Sercu T, Candido S, Rives A. 2023. Evolutionary-scale prediction of atomic-level protein structure with a language model. Science 379:1123–1130. doi:10.1126/science.ade2574 36927031

[28] Rao R, Liu J, Verkuil R, Meier J, Canny JF, Abbeel P, Sercu T, Rives A. 2021. MSA transformer. Proceedings of the 38th International Conference on Machine Learning. Vol. 139, p 8844–8856

[29] Rives A, Meier J, Sercu T, Goyal S, Lin Z, Liu J, Guo D, Ott M, Zitnick CL, Ma J, Fergus R. 2021. Biological structure and function emerge from scaling unsupervised learning to 250 million protein sequences. Proc Natl Acad Sci USA 118:e2016239118. doi:10.1073/pnas.2016239118 33876751 PMC8053943

[30] Chandra A, Tünnermann L, Löfstedt T, Gratz R. 2023. Transformer-based deep learning for predicting protein properties in the life sciences. eLife 12:e82819. doi:10.7554/eLife.82819 36651724 PMC9848389

[31] Dumontet L, Han S-R, Lee JH, Oh T-J, Kang M. 2026. Trustworthy prediction of enzyme commission numbers using a hierarchical interpretable transformer. Nat Commun 17:1146. doi:10.1038/s41467-026-68727-3 41617688 PMC12858967

[32] Han S-R, Park M, Kosaraju S, Lee J, Lee H, Lee JH, Oh T-J, Kang M. 2023. Evidential deep learning for trustworthy prediction of enzyme commission number. Brief Bioinformatics 25:bbad401. doi:10.1093/bib/bbad401 37991247 PMC10664415

[33] Dumontet L, Han S-R, Prouvost A, Lee JH, Oh T-J, Kang M. 2025. Interpretable Kolmogorov-Arnold networks for enzyme commission number prediction. bioRxiv. doi:10.1101/2025.01.30.633071

[34] Chefer H, Gur S, Wolf L. 2021. Transformer interpretability beyond attention visualization. Proceedings of the IEEE/CVF Conference on Computer Vision and Pattern Recognition. p 782–791

[35] García-Vinuesa J, Rojas J, Soto-García N, Martínez N, Alvarez-Saravia D, Uribe-Paredes R, Davari MD, Conca C, Asenjo JA, Medina-Ortiz D. 2026. Geometric deep learning assists protein engineering. Opportunities and challenges. Biotechnol Adv 87:108790. doi:10.1016/j.biotechadv.2025.108790 41456696

[36] Fazel Z, de Souza CPE, Golding GB, Ilie L. 2025. Explainability of protein deep learning models. Int J Mol Sci 26:5255. doi:10.3390/ijms26115255 40508065 PMC12154934

[37] Gambarini V, Pantos O, Kingsbury JM, Weaver L, Handley KM, Lear G. 2022. PlasticDB: a database of microorganisms and proteins linked to plastic biodegradation. Database (Oxford) 2022:baac008. doi:10.1093/database/baac008 35266524 PMC9216477

[38] Camacho C, Coulouris G, Avagyan V, Ma N, Papadopoulos J, Bealer K, Madden TL. 2009. BLAST+: architecture and applications. BMC Bioinformatics 10:421. doi:10.1186/1471-2105-10-421 20003500 PMC2803857

[39] Li W, Godzik A. 2006. Cd-hit: a fast program for clustering and comparing large sets of protein or nucleotide sequences. Bioinformatics 22:1658–1659. doi:10.1093/bioinformatics/btl158 16731699

[40] Fischer M, Pleiss J. 2003. The Lipase Engineering Database: a navigation and analysis tool for protein families. Nucleic Acids Res 31:319–321. doi:10.1093/nar/gkg015 12520012 PMC165462

[41] Vaswani A. 2017. Attention is all you need. *In* Advances in neural information processing systems

[42] Jing X, Dong Q, Hong D, Lu R. 2020. Amino acid encoding methods for protein sequences: a comprehensive review and assessment. IEEE/ACM Trans Comput Biol Bioinform 17:1918–1931. doi:10.1109/TCBB.2019.2911677 30998480

[43] Almeida BC, Figueiredo P, Carvalho ATP. 2019. Polycaprolactone enzymatic hydrolysis: a mechanistic study. ACS Omega 4:6769–6774. doi:10.1021/acsomega.9b00345

[44] Carr PD, Ollis DL. 2009. Alpha/beta hydrolase fold: an update. Protein Pept Lett 16:1137–1148. doi:10.2174/092986609789071298 19508187

[45] Wei R, Zimmermann W. 2017. Biocatalysis as a green route for recycling the recalcitrant plastic polyethylene terephthalate. Microb Biotechnol 10:1302–1307. doi:10.1111/1751-7915.12714 28401691 PMC5658586

