# Supplementary material for: Interpretable convolutional neural networks for sequence-based classification and discovery of plastic-degrading enzymes
Source: Appl Environ Microbiol. 2026 Apr 30;92(5):e01586-25. doi: 10.1128/aem.01586-25 (PMC13188855; doi:10.1128/aem.01586-25)
Supplement: Supplemental material — Notes S1 to S3; Table S1. [file aem.01586-25-s0001.pdf]

Supplementary:

Interpretable Convolutional Neural Networks for Sequence-based Classification and Discovery of Plastic-Degrading Enzymes

Woo-Haeng Lee[1+], Louis Dumontet[2+], KyungMin Jung[3], Hyun Lee[3,4], Gobinda Thapa[1], Tae-Jin Oh[1,4,5*], and Mingon Kang[2*]

[1] Department of Life Science and Biochemical Engineering, SunMoon University, Asan, Republic of Korea 31460

[2] Department of Computer Science at the University of Nevada, Las Vegas, NV, USA 89154

[3] Department of Computer Science and Engineering, SunMoon University, Asan, Republic of Korea 31460

[4] Genome-based BioIT Convergence Institute, Asan, Republic of Korea 31460

[5] Department of Pharmaceutical Engineering and Biotechnology, SunMoon University, Asan, Republic of Korea 31460

[+]The first authors: Woo-Haeng Lee and Louis Dumontet
*Corresponding authors: Tae-Jin Oh and Mingon Kang

Supplementary Note 1. Transformer architecture.

We considered an encoder-only transformer for the benchmark. It is composed of an embedding module followed by four encoders. The embedding module relies on two layers: the amino acid matrix embedding and the positional encoding. The amino acid matrix embedding is a $23 \times d_{model}$ ($d_{model} = 256$) matrix learned during training such that the amino acid (assigned index a) has its embedding in the a-th row of this matrix. Note that we need an embedding for the 20 different amino acids: the undetermined amino acid often referred to X, the classification token, as well as the padding token which account for the 23 rows of our matrix. The positional encoding module takes the position p of the amino acid as input and encodes it as:

$$PE(p, 2i) = sin(\frac{p}{10000^{2i/d_{model}}}), \quad (1)$$

$$PE(p, 2i + 1) = cos(\frac{p}{10000^{2i/d_{model}}}), \quad (2)$$

where 2i and 2i+1 represent even and odd indices, respectively. The outputs of the two layers are then added to form the output of our embedding module, which is fed to the first encoder.

Each encoder comprises a multi-headed attention module followed by two fully connected layers activated by the ReLU function, a dropout layer, and a layer normalization. The multi-headed attention layer consists of projecting the input into 3 vectors Q (query), K (key), and V (value) of size $d_k = 256$. The output of the attention module is computed as:

$$Attention\ Scores = Softmax(\frac{Q \cdot K^T}{\sqrt{d_k}}), \quad (3)$$

$$Attention\ output = Attention\ Scores \cdot K. \quad (4)$$

Then, the output is fed to a dropout layer with a dropout rate of 0.1. The multi-headed attention module incorporates a skip connection, which adds the input of this module to its output. The given result is normalized and fed to two successive position-wise feed-forward layers of size $4 * d_k = 1024$ in which the first one uses ReLU as an activation function, and both layers apply a dropout of 0.1 to their output. These two layers are skipped through a skip connection that adds the module's input to its output.

The final output of the encoder is passed through a dropout layer with a dropout rate of 0.1. After going through the embedding module and the four encoders, the final prediction is made by a linear layer, which takes as input the representation of the classification token by the last encoder of the transformer and uses the sigmoid function as activation to produce a score between 0 and 1 for each of the 11 classes.

Supplementary Note 2. Predictive performance comparison of CNNs across various encoders.

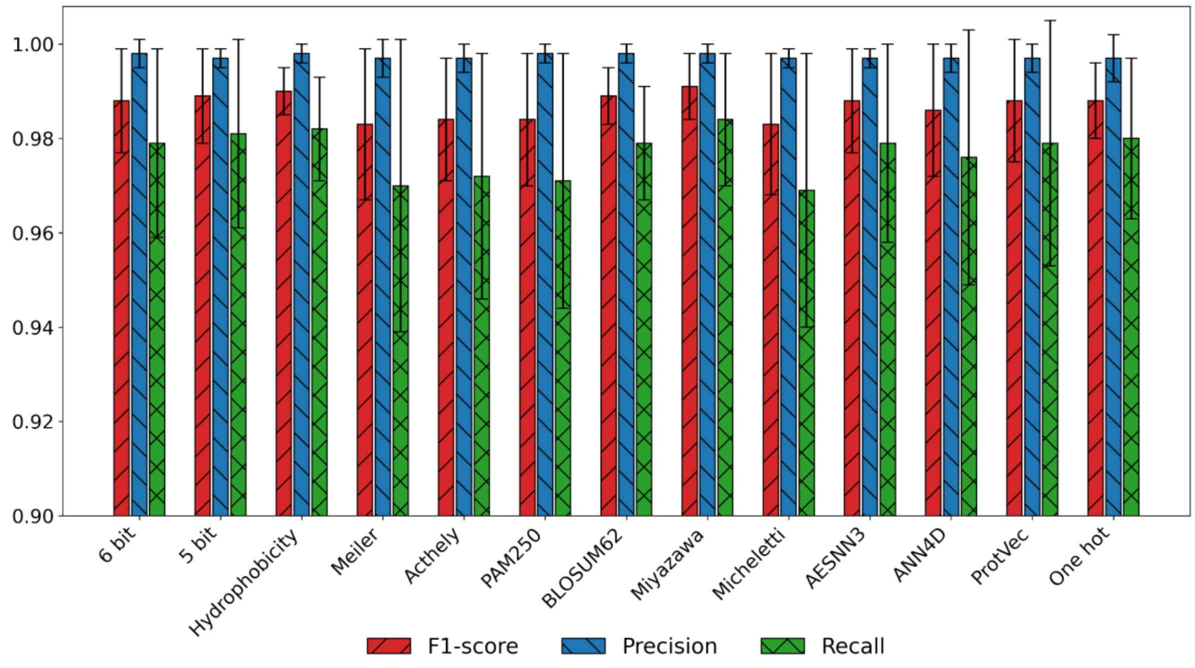

Fig. S1. Predictive performance of convolutional neural networks on the cross-validation dataset across multiple encoding methods

We evaluated the predictive performance of thirteen amino acid encoding methods using PEPIC on the cross-validation dataset (Fig. S1). Performance was assessed on the validation sets using micro-averaged F1-score across twenty repetitions to ensure statistical robustness. Among the tested encodings, Miyazawa energies achieved the highest micro-averaged F1-score (0.991 ± 0.007), followed closely by hydrophobicity matrix (0.990 ± 0.005), binary 5-bit encoding (0.989 ± 0.010), and BLOSUM62 (0.989 ± 0.006). Other encoding methods, such as one-hot (0.988 ± 0.008), AESNN3 (0.988 ± 0.011), and PAM250 (0.984 ± 0.014), also demonstrated competitive performance. Despite slight variations in the results, none of the differences were statistically significant. Given its strong performance, we adopted the Miyazawa energies encoding for the remainder of this study.

Supplementary Note 3. Experimental Validation of Predicted Enzymes

Cloning and Heterologous Expression

The PETase-like gene was amplified by polymerase chain reaction (PCR) using specific primers listed in the Supplementary Data. The 951 bp PCR fragment was ligated into a T-vector and introduced into *E. coli* XL1-Blue for initial cloning. Both the recombinant plasmid and the expression vector pET-32a(+) were subsequently digested with *EcoRI* and *HindIII* and ligated using T4 DNA ligase. Successful ligation and insert integrity were verified by Sanger sequencing using T7 promoter and T7 terminator primers. The confirmed construct was transformed into *E. coli* BL21(DE3) cells for protein expression.

For expression, a single positive colony was cultured in lysogeny broth (LB) supplemented with 100 μg/mL ampicillin at 37 °C until the optical density at 600 nm reached 0.6–0.7. Protein expression was induced with 0.5 mM isopropyl β-D-1-thiogalactopyranoside (IPTG), and the culture was incubated for an additional 24 h at 20 °C. The induced cells were harvested by centrifugation at 3,500 rpm for 30 min at 4 °C, washed twice with 50 mM Tris-HCl buffer (pH 8.0), and stored at −50 °C until purification.

Protein Purification

The frozen cell pellets were resuspended in 8 mL of 50 mM Tris-HCl buffer (pH 8.0) and disrupted by ultrasonication for 15 min using 5 s on/off pulses (Ulsso HiTech, Korea). The lysate was clarified by centrifugation at 11,000 rpm for 25 min at 4 °C. The supernatant containing soluble protein was loaded onto a gravity-flow affinity column pre-equilibrated with Tris-HCl buffer, washed with 20 mL of the same buffer, and eluted with an imidazole-containing buffer. The eluted fraction was concentrated using Amicon Ultra centrifugal filters (30 kDa cutoff; Millipore). Protein purity was assessed by 15% SDS-PAGE, and the concentration was determined by the Bradford assay.

Enzymatic Activity Assay

Purified enzyme (50 nM) was incubated in 500 μL of 50 mM Tris-HCl buffer (pH 7.0) containing 4 mM bis(2-hydroxyethyl) terephthalate (BHET) at 30 °C for 18 h. The reaction was stopped by heating at 80 °C for 10 min and filtered through a 0.22 μm syringe filter (Whatman, Germany).

Negative controls were prepared using heat-denatured enzymes, and all assays were performed in triplicate.

HPLC analysis was carried out on a Dionex UltiMate 3000 DAD system (Thermo Fisher Scientific, Japan) using a Mightysil RP C18 column (4.6 × 250 mm, 5 μm). The mobile phases were (A) 0.05% TFA in water and (B) acetonitrile. A gradient of 30–65% B (0–25 min) followed by 65–100% B (25–35 min) was used at a flow rate of 1.0 mL/min. The injection volume was 20 μL, the column temperature was maintained at 30 °C, and detection was performed at 260 nm.

Supplementary Table 1. Sequences for interpretation of plastics-hydrolyzing enzymes.

Class	Accession number (Nr)	Sequence
PET	MAA58622.1	MNTYLLRTLSICLFAGLFSMQVQAITPDPEPDPDPDPPSTCSNCYQRGPNPTVSALEADSGPYSVRTINVSSWVSGFGGGTIHYPVGTEGTMGAIAVIPGYVSYERSIKWWGPRLASWGFVVITTDTNTIYDQPDSRADQLSAALDYVISQSNSSRSPIYGMVDANRLGAMGWSMGGGGTLKLSTERELKAAIPQAPYYAGFNPFDEITTPTLIIACELDVVAPVAQHASPFYREIPGSTAKAFLEINGGDHFCANSGYPDEDILGKYGIAWMKRFIDEDRRYDQFLCGPNHEADRSISEYRDTCNY
	RLP53020.1	MLEGRYMKTVRFNTAAAVFTSALLSSQVFAITDDPVDPVDPVDPPSSGTVRGPDPTLSALESTRSGPYSVRTENVSNLSASGFGGGTIHYPTNAGENMGAIAVIPGYVSYESSIEWWGPRLASWGFVVITIDTNTIYDQPDSRADQLSAALDHLIDESGSSSSPISGLVDASRLGVIGWSMGGGGTLKLATERNLKAIIPQAPWYSGFNSFDRITTPTMIIACESDAIAPVGQHASPFYNDIPNSTAKAFLEINGGSHYCANSGYSDEDILGKYGISWMKRFMDNDTRYSQFLCGPNHESDRSISEYRDTCNY
	WP_021018894.1	MNVLTKCKLALGIIAIFFSLPSFAVPCSDCSNGFERGQVPRVDQLESSRGPYSVKTINVSRLARGFGGGTIHYSTESGGQQGIIAVVPGYVSLEGSIKWWGPRLASWGFTVITIDTNTIYDQPDSRASQLSAAIDYVIDKGNDRSSPIYGLVDPNRVGVIGWSMGGGGSLKLATDRKIDAVIPQAPWYLGLSRFSSITSPTMIIACQADVVAPVSVHASRFYNQIPGTTPKAYFEIALGSHFCANTGYPSEDILGRNGVAWMKRFIDKDERYTQFLCGQNFDSSLRVSEYRDNCSYY
	WP_077315388.1	MKFLIKVNFILSIFAVFISPQIFAATVACSDCSNGFQRGTLPRVDQLESSRGPYSVKTSNVSVFARGFGGGTIHYSTDSGGQQGIIAVIPGYVSYESSIKWWGPRLASWGFTVITINTNTIYDQPNSRANQLSAAIDYVIDKGNDRSSPIYGLVDPERVGVIGWSMGGGGTLKLATDRDIDAIIPQAPWYSGLNSFSRITTPTMVIACQADAVAPVALHASIFYNQIPRSTPKAFFEIAAGSHFCGNSGYPNEDILGRNGVAWMKRFIDNDTRYNQFLCGQNFDRSLRVSDYKDTCNTY
	RMH89651.1	MKLKAYLARITSLVTVSLLASSLAYAAPGPSAPCADCSRGPNPTVASLQSRSGPFTVSTFSVSGYLRGFGNSTVHYPTNATGKMGAIAVIPGYLSYEDSIRWWGPRLASHGFVVITMNTNTIYDQPDSRATQLSRALDYVIEQSNSRSSPISGKVDSTRLGAIGWSMGGGGSLKLSTERSLNAIIPQAPYYAGLNRFNTINTPTMIL

		ACSADVVAPVGSHASPFYNRIPEATPKAFLEIYGGSHFCANSGYPNEDLLG<br>MYGISWMKRFIDFDSRYSQFLCGPNHAADLRISEYRENCNY
	OUS39971.1	MPNPNPAPCQEDCDFTRGPDPTISSLEASAGPYSVANQGVSRSVDGFGGGTI<br>FYPMNTTGTMGAIAIAPGFLAGESSIEWWGPRFASHGFVIITIATNSVFDQPN<br>SRETQLSSALDYVISQSNSGNSPISGMVDSTRVGAMGWSMGGGGALRLAS<br>GDRLSAVIPLAPWHQGRNSFDQLETPTLIIACENDTVAPVNRHASSFYNSIPS<br>STDKALLEISNGAHSCANGGGANGGLLGKYGVSWMKRFIDNDLRYDQFLC<br>GPNHAANSAVSEYRGTCNY
	WP_0859886<br>67.1	MPLSMNKLQKLPLTLTSAALLCAGGLTVNTAVAETRGPDPTEAYVEAEGP<br>YNVDTINVSSLALGFGGGTIHYPTNTTGQMGGIVVIPGYLSYESSIEWWGER<br>LASHGFVVMTIDTNTIYDQPGSRRDQIDAALDYLVDRSDSSFSAISGMVDG<br>DRLGAVGWSMGGGGTLQLASGDRLSAAIPLAPWNSSFNDFDDIETPTLIFA<br>CENDTVAPVGVHASPFYYDIPASTDKAFFEINNGNHFCANGDNANDAVLS<br>KYGVSWMKLHIDQDARYGQFLCGPNHESQYRISEYRGTCPY
	MBF78136.1	MPFNKKGILAACGAGALLFSMSALANNPPPTDPDPGDGGSSPYQRGPDPSV<br>SFLEADRGNYSVSTSRVSGLVSGFGGGTIHYPSGTTGTMAAIVVIPGFVSAE<br>SSIEWWGPKLASYGFVVMTIDTNSGFDQPGSRATQINNALDYLVSQNTSSS<br>SPVRGMIDTSRLGVVGWSMGGGGTLRVAREGRIKAAIPLAPWDTSTYYSS<br>RSQAPTLIFACESDVIAPVYQHASPFYNALPSSIDKAFVEINNGSHYCGNGG<br>SIYNDVLSRFGVSWMKLHLDEDARYKQFLCGPNHTSDSQISDYRGNCPY
	WP_1181318<br>88.1	MSALANNPPPTDPDPGNGGSSPYQRGPDPSVNFLEADRGQYNVDDERVSSF<br>VSGFGGGTIHYPTGTTGTMAAIVVIPGFVSAESSIEWWGPKLASYGFVVMTI<br>DTNSGFDQPGSRATQINNALDYLVDQNTSVGSPVRGMIDTDRLGVIGWSM<br>GGGGTLRVGREGRIKAAIPLAPWDTSSYYASRAQAPTLIFACESDVIAPVYQ<br>HASPFYNALPSNIDKAFVEINNGSHYCGNGGSIYNDVLSRFGVSWMKLHLD<br>EDARYKQFLCGPNHTSDSQISDYRGNCPY
	WP_0918498<br>96.1	MSDHYASNPLRSVVAAASLLFSASVFAAGGGGSDGGDDGCTSNCGYERGP<br>APTESFLEASSGPYSVRTDRVSSLVGGFGGGTIHYPTGTSGTMGAVVVIPGF<br>VSAESSIEWWGPKLASHGFVVMTIDTNSGFDQPPSRATQINNALDYLIEQN<br>GSSSSPVSGMIDTNRLGVIGWSMGGGGTLRVASEGRIQAAIPLAPWDTSSFR<br>FRNIETPTLIIACESDIIAPVRSHADPFYEAIPSSTDKAFVELNNGSHYCGNGG<br>NSYNDVLSRFGVSWMKLHLDNDQRYNQFLCGPDHERDWDISEYRGTCPY
	CAH17554.1	MAVMTPRRERSSLLSRALRFTAAAATALVTAVSLAAPAHAANPYERGPNP<br>TDALLEARSGPFSVSEERASRFGADGFGGGTIYYPRENNTYGAVAISPGYTG<br>TQASVAWLGKRIASHGFVVITIDTNTTLDQPDSRARQLNAALDYMINDASS<br>AVRSRIDSSRLAVMGHSMGGGGSLRLASQRPDLKAAIPLTPWHLNKNWSS<br>VRVPTLIIGADLDTIAPVLTHARPFYNSLPTSISKAYLELDGATHFAPNIPNKI<br>IGKYSVAWLKRFVDNDTRYTQFLCPGPRDGLFGEVEEYRSTCPF
	G8GER6.1	MPPHAARPGPAQNRRGRAMAVITPRRERSSLLSRALRFTAAAATALVTAVS<br>LAAPAHAANPYERGPNPTDALLEARSGPFSVSEERASRFGADGFGGGTIYY<br>PRENNTYGAVAISPGYTGTQASVAWLGERIASHGFVVITIDTNTTLDQPDSR<br>ARQLNAALDYMINDASSAVRSRIDSSRLAVMGHSMGGGGTLRLASQRPDL<br>KAAIPLTPWHLNKNWSSVRVPTLIIGADLDTIAPVLTHARPFYNSLPTSISKA<br>YLELDGATHFAPNIPNKIIGKYSVAWLKRFVDNDTRYTQFLCPGPRDGLFG<br>EVEEYRSTCPF
	WP_1247733<br>20.1	MSSPTTTRPRSVVARLALAAVLAAGGVLAGPAGVAQAASPYERGPAPTTAI<br>LEASRGPFATASQSVSSLSVVGFGGGVIYYPTSTSEGTFGAIAISPGFTASWS<br>SISWLGPRIASHGFVVIGIETNTRLDQPDSRGRQLLAALDYLTERSSVRSRID<br>SSRLAVAGHSMGGGGSLEAASSRPSLQAAVPLAPWNTDKSWSELRVPTLII<br>GGESDSVAPVATHSVPFYNSIPASAEKAYLELNGASHFFPQTTNTPTARQM<br>VAWLKRFVDDDTRYEQFLCPGPSGSQIQEYRNTCPSA

	TKK88911.1	MSRIATAALAFTLATGTAVTLAPSAQAAGFERGPNPTSAILEASRGPFSVATTSVSSLVSGFGGGTIYYPTDTSQGTFGAIAISPGYTARWSSLEWLGPRIASHGFVVIGIETNSTLDQPASRGNQLLAALDYLVNSSSTTVRSRIDRNRLAVAGHSMGGGGTLHAAEDRPSLKAAVPIAPWNTDKTWGSVRVPTLIVAGESDSVASPTTHASPFYNSITQTEKAYLELNSASHFFPQTTNTPFAKQFVAWLKRWVDEDTRYSQFICPGPSGLAIEEYRSTCPV
	WP_160875656.1	MTTTTWRTRIASLALAAAAATGLTSGVGLGAPISAVAATANPYERGPAPTRASIEATRGAYATAQTSVSSLVSGFGGGTIYYPTTTADGTFGAVIIAPGYTATSSSLAWLGPRLASQGFVVFTIDTDSRYDQPASRGDQLLEAADYLTRTSVVASRVDARRVALMGHSMGGGGTLEAIKDRPSIKAAIPLTPWNLDKTWPEVTTPTLIIGADNDSVAPVASHAEPFYGSLPSTLDKAYLELRNASHFAPNSANTTIASYSIAWLKRFVDDDTRYSQFLCPTPASSLAIAEYRSTCPY
	WP_142569056.1	MVTRLALVVLLALAGGLLTAPAAHAAVHGPDPTDALLESSRGPYATAQTDVSSLSVSGFGGGTIYYPTTTSEGTFGGVAIAPGYTADKSSLAWLAARLASHGFVVFNIDTLTRLDQPDSRGRQLLAALDYLTQRSSVRGRVDATRLGVMGHSMGGGGTLEAVDDRPSVRAAVPLTPWNLDKTWSGVRTPTLIIGAEADTVAPVASHAVPFYTSLSSSLDKAYLELNGATHFAPNTTNTTIGKYAVAWMKRFVDDDTRYDQFLCPGPGRSLTVEEYRSTCPF
	EFL43114.1	MHRPAGGSPRQRGPLVVQQHPHTGGRRTGRFAALAAAVAAVVGLTTLGGPGAHAADNPYERGPAPTESSIEALRGPYAVSETSVSSLSVTGFGGGTIYYPTSTADGTFGAIAVSPGFTAYQSSIAWLGPRLASQGFVVFTIDTNTTLDQPASRGDQLLAALDYLTQRSAVRGRIDSSRLGVMGHSMGGGGTLEAAKDRPSLQAAIPLTPWNLDKTWPEVRTPTLLFGADGDTVAPVGTHAEPLYTGLPSSLDRAYLELNGATHFTPNSSNTTIAKYSISWLKRFIDNDTRYEQFLCPLPRPSLTIEESRGNCPHTS
	WP_085576151.1	MQQHPHTSRRGTGRFAALTAAVAAVVGLTTLNGPGAQAADNPYERGPAPTESSIEALRGPYAVSDVSVSSLSVTGFGGGTIYYPTSTADGTFGAIAISPGFTAYQSSIAWLGPRLASQGFVVFTIDTNTTLDQPASRGDQLLAALDYLTQRSAVRGRVDSSRLGVMGHSMGGGGTLEAAKDRPSLQAAIPLTPWNLDKTWPEVRTPTLLFGADGDTVAPVSSHAEPLYSGLPSSLDRAYIELNGATHFTPNSSNTTIAKYSVSWLKRFIDNDTRYEQFLCPLPRPSLTVEESRGNCPHTS
	WP_150474004.1	MQQQARTGAAHRVPTGSPHRRSARFAGLATAIAAVVGLTTLNGAGAQAADNPYERGPAPTTSSIEAARGSYSVSQTSVSSLAVTGFGGGTIYYPTSTADGTFGAVAISPGYTGTQSTMAWLGPRLASQGFVVFTIDTNTTLDQPDSRGRQLLAALDYLTQRSSVRGRVDSTRLGVMGHSMGGGGSLEAAKTRPSLQAAIPLTPWNTDKSWPEISTPTLIFGADGDTIAPVASHAEPFYSSLPSSLDRAYLELNGTSHLTPISSNTTIAKYSVSWLKRFIDNDTRYEQFLCPLPRPSLTIEEYRGNCPHTS
	WP_053757025.1	MQQHPRSTTASAAPGPARGAGRRGTRRFAGAAAAIAAAVALSTLTGPGARAADNPYERGPAPTTASIEASRGPYSVSETSVSSLAVSGFGGGTIYYPTSTADGTFGAVAVSPGYTGTQSSIAWLGPRLASQGFVVFTIDTLTTLDQPDSRGRQLLAALDYLTTRSSVRGRVDSTRLGVMGHSMGGGGSLEAAKSRPSLQAAIPLTPWNLDKSWPEVTTPTLIVGADGDSIAPVSSHAEPFYGSLRSSLDRAYLELNGASHFTPNSSNTTIAKYSVSWLKRFIDNDTRYEQFLCPLPSPSLTIEEYRGNCPHTS
	BCL25765.1	MQQHSQNSSLTAGTVPPESAGRRPRRRDGRGGWAAKRITGALAALTTVVGLSSLASPGAHAADNPYERGPAPTTSSIEAARGSYSVSQTTVSSLAVTGFGGGTVYYPTSTADGTFGAVVISPGYTGTQSSISWLGARLASQGFAVFTIDTLTTLDQPDSRGRQLLAALDYLTERSSVRTRVDGSRLAVMGHSMGGGGSLEAAKSRPSLQAAIPLTPWNTDKSWPEVSTPTLIVGADGDTIAPVASHAEPFYGSLPSSTDKAYLELNNATHFTPNSSDTTIAKYSISWLKRFVDNDTRYEQFLCPLPRPSLTIEEYRGNCPHTS

	WP_069933776.1	MRNAPAHRRRGSGRLRSLVAGLAALLAVGGLSSVATPAAQAADNPYERGPAPTTASIEAPNGPYAVSQTSVSSLSVTGFGGGTVYYPTTTGDGTFGAVAISPGFTAGESSIAWLGPRLASQGFVVFTIGTLTRYDQPDSRGSQLLAALDYLTQRSTVRARIDSGRLGVMGHSMGGGGSLEAAKSRPSLQAAIPLTGWNTDKTWPEIKTPTLVVGADGDTVASVGSHSEPFYESLPSSLDKAYLELNNATHFTPNTSNTTIAKYSISWLKRFIDNDTRYEQFLCPLPRPSLTIEEYRGNCPHTS
	WP_237325367.1	MQQHLLARRQTPHPSRSRTLTGLLTAAAATAGLLLTALAPGAQAVAANPYERGPAPTNASIEASRGSYATSQTSVSSLAVSGFGGGTIYYPTSTADGTFGAVVISPGFTAYQSSIAWLGPRLASQGFVVFTIDTNTTLDQPDSRGRQLLSALDYLTQRSSVRTRVDASRLGVMGHSMGGGGSLEAAKSRTSLKAAIPLTGWNTDKTWPELRTPTLVVGADGDTVAPVGTHSKPFYESLPGSLDKAYLELRGASHFTPNSSDTTIAKYSLSWLKRFIDNDTRYEQFLCPIPRPSLTIAEYRGTCPHTS
	WP_161267637.1	MQQHLLARRQAPRPSRSRTLTGLLTAAAATAGLLLTGLAPGAQAVAANPYERGPAPTNASIEASRGSYATSQTSVSSLAVSGFGGGTIYYPTSTADGTFGAVVISPGFTAYQSSIAWLGPRLASQGFVVFTIDTNTTLDQPDSRGRQLLSALDYLTQRSSVRTRVDASRLGVMGHSMGGGGSLEAAKSRTSLKAAIPLTGWNTDKTWPELRTPTLVVGADGDTVAPVATHSKPFYESLPGSLDKAYLELRGASHFTPNSSDTTIAKYSLSWLKRFIDNDTRYEQFLCPIPRPSLTIAEYRGTCPHSS
	AOS64284.1	MQSSSIASRRARVRSAGRPRTRLAGLVLALTMVATGLAAAPAATAQENPYERGPAPTERSIEALRGPFAVAEDRVSSLVIGFGGGTIYYPTDTSEGTFGAVAVSPGYTGTQSSMAWLGPRLASQGFVVFTIDTNTTVDQPDSRGRQLLAALDYLVEDSDVRNRIDPNRLGVMGHSMGGGGSLSAAESRPALQAAIPLTGWHLSKNWSRVTVPTLVVGAENDLIAPVRSHSIPFYESLSSSLDKAYLELDGASHFAPNISNTTIAKYSISWLKRFIDDDLRYEQFLCPPPDDREISEYRNTCPHS
	KOV83888.1	MPNEVYSAVQLRTLIPLALTLVLVTGTAAQAADNPYERGPAPTVSSIEALRGPFAVSETSVSSLVGGFGGGTIYYPTSTTSGTFGAVAVSPGYTGTQSSISWLGPRLASQGFVVFTIDTNTIYDQPDSRASQLLAALDYLTQQSSVRSRIDATRLGVMGHSMGGGGTLRAASQRPTLQAAIPLTAWHTTKNWSSVRVPTLVVGAEDDSIAPVATHSEPFYTTLPSTLDKAYLELNNATHFAPNSNNTTIAKYSISWLKRFIDNDTRYEQFLCPAPGRSTLIEEYRDTCPHS
	WP_373873195.1	MDKVIPKLFGIAAAVALGGAGITLIPDADAATASFAKGPAPSNASIEAVRGPFAVAQSNVSRASVSGFGGGDIYAPTDTGAGTFGAVVIAPGFTARKSSMAWLAPRLASQGFVVFNIDTLSTSDQPASRGRQLLAAADFLTQRSTVRARIDAGRVAVIGHSMGGGGALEAAGSRPALAAAIPLTPWNLTKSFSRNAVPTLVIGAEADSIAPVRSHAQPFFQSLPAVPGKAFLNLNGASHFAPNTPNTTIAKFSISWLKLFVDDDTRYQQFVCPGPGAGAAVQEYRSTCDQFTP
	WP_328594753.1	MPRTTLRTLAAAVLAAGAVGVLPTAPAHAAGFERGPAPTEASVTAAKGPFAIDRIEVPAGSGTGFNSGTIYYPTSTAEGTFGAVAISPGFVSPKSWIDWYGPRLASQGFVVMTLETFSYFDAPDGRADQLLAALDYLTAKSKVKDRIDPNRLAAMGHSMGGGGALSAAVKRPSLKAVVPLAPWYVGGGLEQSTVPTMIFGADNDFIAPVASNARPFYQSLTKVPEKAYLELENAGHVGSFNSPNTTIAKYAISWLKRFVDDDTRYSQFLCPAPKFPSSTIQEYRDTCPS
	G9BY57.1	MDGVLWRVRTAALMAALLALAAWALVWASPSVEAQSNPYQRGPNPTRSALTADGPFSVATYTVSRLSVSGFGGGVIYYPTGTSLTFGGIAMSPGYTADASSLAWLGRRLASHGFVVLVINTNSRFDYPDSRASQLSAALNYLRTSSPSAVRARLDANRLAVAGHSMGGGGTLRIAEQNPSLKAAVPLTPWHTDKTFNTSVPVLIVGAEADTVAPVSQHAIPFYQNLPSTTPKVYVELDNASHFAPNSNNAAISVYTISWMKLWVDNDTRYRQFLCNVNDPALSDFRTNNRHCQ
	P19833.1	MFIMIKKSELAKAIIVTGALVFSIPTLAEVTLSETTVSSIKSEATVSSTKKALPATPSDCIADSKITAVALSDTRDNGPFSIRTKRISRQSAKGFGGGTIHYPTNASGCGLLGAIAVVPGYVSYENSIKWWGPRLASWGFVVITINTNSIYDDPDSRAAQLNAALDNMIADDTVGSMIDPKRLGAIGWSMGGGGALKLATERSTVRAI

		MPLAPYHDKSYGEVKTPTLVIACEDDRIAETKKYANAFYKNAIGPKMKVE<br>VNNGSHFCPSYRFNEILLSKPGIAWMQRYINNDTRFDKFLCANENYSKSPRI<br>SAYDYKDCP
PCL	WP_025804184.1	MSTLSWVRGVNGTLGWVAPKLVASKMRLAFMTPREHLPRDWELPLLARS<br>ERITLRFGLSALRWGQGPAVLLMHGWEGRPTQFASLIDALVGAGYSVVAL<br>DGPAHGRSPGHEANVMLFARAMLEAAAELPPLRAVIGHSMGGASAMLAV<br>QLGLRTETLVSIAAPARILGVLRGFARYVRLPPKARSVFIRQVEQDVGMRA<br>AAMDVAHYQLDMPGLIVHAEDDNFVPVKESELIHDAWFDSRLLRLKEGG<br>HQRVLADPRVIEGVLTLLAGRSLQARQSA
	WP_059181212.1	MSTLKWVRGVNGTLGWFAPQLVASRMRLAFMSPRALPPRDWELPLLAKS<br>ERITLRFGLSALRWGQGPAVLLMHGWEGRPTQFASIITALVDAGYSVVALD<br>GPAHGRSPGEEANVVLFARAMLEAAAELPPLQAVIGHSMGGASAMLAVQ<br>LGLRTETLVTIAAPARILGVLRGFAKYVRLPPKARSAFIRQVEKDVGMRAA<br>ALDVAHYQLDMPGLIVHAEDDRFVSAKESQLIHEAWFDSRLLRLEEGGHQ<br>RVLADPRVIDGVLSLLAGRSLHSRQSA
	WP_052145136.1	MERLYRGMGRAALDRAYNNTRAIANFPAVLADFRTRSAALYERVRGRRD<br>LRYGDRPRERFDWLPGGRANAPTFVFIHGGYWQNCAKEDFAFVAHGPLAR<br>GFNVVLAEYTLAPDASMTQIVDEIGRLIDHLRADRDGLGTAGRPLCLSGHS<br>AGGHLSALHRGHAFVTSALAISPLVDLEPISLSWLNEKLQLSEREIAAYSPL<br>WHVGKGAPTVVAVGADELPELVRQADDYTAACAAAGEPVWGAHVPGCT<br>HFSVLDDLAQPNGTLMRLLDAAIAGSGHGGDERADEERR
	WP_124320071.1	MSTLSWVRGVNGTLGWVAPKLVASKMRLAFMTPRERLPRDWELPLLARS<br>ERITLRFGLSALRWGQGPAVLLMHGWEGRPTQFASLIDALVGAGYSVVAL<br>DGPAHGRSPGQEANVMLFARAMLEAAAELPPLRAVIGHSMGGASAMLAV<br>QLGLRTETLVSIAAPARILGVLRGFARYVRLPPKARSAFIRQVEQDVGMRA<br>AAMDVAHYQLDMPGLIVHAEDDNFVPVKESDLIHEAWFDSRLLRLKEGG<br>HQRVLADPRVIEGVLTLLAGRSLQARQSA
	WP_134174323.1	MNTLKWIRGVNGTLGWIAPKRVASKMRLAFMTPRSLPLRDWELPLLASSE<br>RITLRFGLSALRWGQGPAVLLMHGWEGRPTQFAALITALVDAGYTVVALD<br>GPAHGRSPGREANVVLFARAMLEAAAELPPLQAVIGHSMGGASAMLAVQ<br>LGLRTETLVSIAAPARILGVLRGFARYVGMPPRARSAFIRQVEQDVGMRAA<br>TLDVAHYQLDMPGLIVHAEDDNFVSVKESQLIHESWFDSRLLRLEGGGHQ<br>RVLADPRVIDGVLSLLAGRSLQARQSA
	WP_096796149.1	MNTLKWVRGVNGTLGWIAPQRVASKMRQAFMTPRTLPLRDWELPLLASA<br>ERITLRFGLSALRWGQGPAVLLMHGWEGRPTQFAALITALVEAGYTVVAL<br>DGPAHGRSPGREANVVLFARAMLEAAAELPPLQAVIGHSMGGASAMLAV<br>QLGLRTETLVSIAAPARILGVLRGFARYVGMPPRARSAFIRQVEQDVGMRA<br>ATLDVAHYQLDMPGLIVHAEDDTFVSVKESQLIHESWFDSRLLRLESGGHQ<br>RVLADPRVVDGVLSLLAGRSLQARQSA
	WP_054596612.1	MSTLSWVRGVNGTLGWVAPQWVASKMRSVFMTPRELPPRDWEMPLLAK<br>SERITLRFGLSALRWGQGPTVLLMHGWEGRPTQFASLITALVDAGYTVVAL<br>DGPAHGRSPGREANVVLFARAMLEAAAELPPLQAVIGHSMGGASAMLAV<br>QLGLRTETLVSIAAPARILAVLRGFARHVRMPPKVRSAFIRKVERDVGIQAS<br>RLDVAHYQLDMPGLIVHAEDDVFVSVNESQLIHDAWFDSRLLRLEEGGHQ<br>RVLADPRVIEGVLSLLSGRSLQARQSA
	WP_123592831.1	MNTLKWVRGVNGTLGWIAPQRVASKMRLAFMTPRSLPLRDWELPLLASS<br>ERITLRFGLSALRWGQGPTVLLMHGWEGRPTQFAALITALVDAGYTVVAL<br>DGPAHGRSLGREANVVLFARAMLEAAAELPPLQAVIGHSMGGASAMLAV<br>QLGLRTETLVSIAAPARILGVLRGFARYVGMPPRARSAFIRQVEQDVGMRA<br>ATLDVAHYQLDMPGLIVHAEDDNFVSVKESQLIHESWFDSRLLRLEGGGH<br>QRVLADPRVVDGVLSLLAGRSLQARQSA

	WP_059728396.1	MTILYRGMDRAALDAAYLNTKVVPDFPALLASMQARSAALYDTAHGRRD<br>LRYGAQPAQRFDWLSCGRPDAPLFVFIHGGYWQHCAKEDFAYAASGPLAR<br>GFDVILAEYTLAPVATMTGIVGEIGALLDYLANDPDAIGTAGRPIHLSGHSA<br>GGHLTAVYRAHPAVVSALAISPLVDLEPISLCVLNDKLQLGAREVDAYSPL<br>RHVGPGAPTVVAVGDAELPELVRQARDYATACEAAGERVVHVGLPGMRH<br>FDVLDDLAKPDGAMLAALQSIAPR
	WP_038631269.1	MSTLSWVRGVNGTLGWVAPTLVASKMRLAFMTPRERLPRDWELPLLARS<br>ERITLRFGLSALRWGQGPAVLLMHGWEGRPTQFASLIDALVGAGYSVVAL<br>DGPAHGRSPGHEANVMLFARAMLEAAAELPPLRAVIGHSMGGASAMLAV<br>QLGLRTETLVSIAAPARILGVLRGFARYVRLPPKARSAFIRQVEQDVGMRA<br>AAMDVAHYQLDMPGLIVHAEDDNFVPVKESDLIHEAWFDSRLLRLKEGG<br>HQRVLADPRVIEGVLTLLAGRSLQARQSA
	AIC18917.1	MTMTLLYRDMNQAQLDAAYNNTQAVPDFPGIYAALQARSASFYASAAGR<br>LNLPYGTAPRQRYDWLPCGKADAPTLIFIHGGYWQNCSKEDFAFIAAGPLA<br>AGFNIVLAEYTLAPQASMTQIVSEIGSLLEHLQADADQLGIAGHKVVLSGH<br>SAGGHLALQFRSHPWVTDVLAISALVDLEPISLSWLNEKLSLSEAEIDAYSP<br>LYHIDKGANTWVAVGADELSELVRQSDEYAKQALARGESVQLIHVPGCTH<br>FSVLDEMAKPQGALLQALSSIR
	WP_139055557.1	MNTLKWVRGVNGTLGWIAPQRVASKMRQAFMTPRTLPLRDWELPLLASS<br>ERITLRFGLSALRWGQGPAVLLMHGWEGRPTQFAALITALVEAGYTVVAL<br>DGPAHGRSPGREANVVLFARAMLEAAAELPPLQAVIGHSMGGASAMLAV<br>QLGLRTETLVSIAAPARILGVLRGFARYVGMPPRARSAFIRQVEQDVGMRA<br>ATLDVAHYQLDMPGLIVHAEDDTFVSVKESQLIHESWFDSRLLRLESGGHQ<br>RVLADPRVVDGVLSLLAGRSLQARQSA
	WP_103304852.1	MNTLKWIRGVNGTLGWIAPKQVASKMRLAFMTPRALPLRDWELPLLANS<br>ERITLRFGLSALRWGQGPTVLLMHGWEGRPTQFAALITALVEAGYTVVAL<br>DGPAHGRSPGREANVVLFARAMLEAAAELPPLQAVIGHSMGGASAMLAV<br>QLGLRTETLVSIAAPARILGVLRGFARYVGMPPRARSAFIRQVEQDVGMRA<br>ATLDVAHYQLDMPGLIVHAEDDNFVSVKESQLIHESWFDSRLLRLESGGH<br>QRVLADPRVIDGVLSLLAGRSLQARQSA
	WP_007969226.1	MNTLKWVRGVNGTLGWIAPKQVASKMRTAFMTPRALPLRDWELPLLASS<br>ERITLRFGLSALRWGQGPAVLLMHGWEGRPTQFAALITALVEAGYTVVAL<br>DGPAHGRSPGREANVVLFARAMLEAAAELPPLQAVIGHSMGGASAMLAV<br>QLGLRTETLVSIAAPARILGVLRGFARYVGMPPRARSAFIRQVEQDVGMRA<br>ATLDVAHYQLDMPGLIVHAEDDTFVSVKESQLIHESWFDSRLMRLESGGH<br>QRVLADPRVVDGVLSLLAGRSLQARQSA
	WP_047600195.1	MNTLKWVRGVNGTLGWIAPQRVANKMRQAFMTPRTLPLRDWELPLLASS<br>ERITLRFGLSALRWGQGPAVLLMHGWEGRPTQFAALITALVEAGYTVVAL<br>DGPAHGRSPGREANVVLFARAMLEAAAELPPLQAVIGHSMGGASAMLAV<br>QLGLRTETLVSIAAPARILGVLRGFARYVGMPPRARSAFIRQVEQDVGMRA<br>ATLDVAHYQLDMPGLIVHAEDDNFVSVKESQLIHESWFDSRLLRLESGGH<br>QRVLADPRVVDGVLSLLAGRSLQARQSA
	WP_130927896.1	MNALKWVRGVNGTLGWFAPKLVASKMRLAFMTPRALPLRDWELPLLASS<br>ERITLRFGLSALRWGQGPTVLLMHGWEGRPTQFASLINALVDAGYTVVAL<br>DGPAHGRSPGREANVVLFARAMLEASAELPPLQAVIGHSMGGASAMLAV<br>QLGLRTETLVSIAAPSRILGVLRGFARMVGMPPRARSAFIRQVEQDVGMRA<br>ATLDVAHYQLDMPGLIVHAEDDNFVSVKESQLIHEAWFDSRLLRLESGGH<br>QRVLADPRVIDGVLSLLAGRSLQARQSA
	WP_123463437.1	MNTLKWIRGVNGTLGWIAPKRVASKMRLAFMTPRTLPLRDWELPLLANSE<br>RITLRFGLSALRWGQGPTVLLMHGWEGRPTQFAALITALVEAGYTVVALD<br>GPAHGRSPGREANVVLFARAMLEAAAELPPLQAVIGHSMGGASAMLAVQ<br>LGLRTETLVSIAAPARILGVLRGFARYVGLPPRARSAFIRQVEQDVGMRAA

		TLDVAHYQLDMPGLIVHAEDDTFVSVKESQLIHESWFDSRLLRLESGGHQR VLADPRVVDGVLSLLAGRSLQARQSA
	WP_151551651.1	MNALKWVRGVNGTLGWFAPKLVASKMRLAFMTPRALPLRDWELPLLASS ERITLRFGLSALRWGQGPTVLLMHGWEGRPTQFASLINALVDAGYTVVAL DGPAHGRSPGREANVVLFARAMLEAAAELPPLQAVIGHSMGGASAMLAV QLGLRTETLVSIAAPARILGVLRGFARMVGMPPRARSAFIRQVEQDVGMRA ATLDVAHYQLDMPGLIVHAEDDNFVSVKESQLIHEAWFDSRLLRLESGGH QRVLADPRVIDGVLSLLAGRSLQARKSA
	WP_123448644.1	MNTLKWVRGVNGTLGWIAPKRVASKMRLAFMTPRSLPLRDWELPLLASS ERITLRFGLSALRWGQGPTVLLMHGWEGRPTQFAALITALVDAGYTVVAL DGPAHGRSPGREANVVLFARAMLEAAAELPPLQAVIGHSMGGASAMLAV QLGLRTETLVSIAAPARILGVLRGFARYVGMPPRARSAFIRQVEQDVGMRA ATLDVAHYQLDMPGLIVHAEDDSFVSVKESQLIHEAWFDSRLLRLEGGGH QRVLADPRVIDGVLSLLAGRSLQARQSA
	WP_127650388.1	MNTLKWVRGVNGTLGWIAPKRVASKMRLAFMTPRALPLRDWELPLLASS ERITLRFGLSALRWGQGPAVLLMHGWEGRPTQFAALITALVEAGYTVVAL DGPAHGRSPGREANVVLFARAMLEAAAELPPLQAVIGHSMGGASAMLAV QLGLRTETLVSIAAPARILGVLRGFARYVGMPPRARSAFIRQVEQDVGMRA ATLDVAHYQLDMPGLIVHAEDDTFVSVKESQLIHESWFDSRLLRLESGGHQ RVLADPRVVDGVLSLLAGRSLQARQSA
	WP_160057137.1	MNTLKWVRGVNGTLGWIAPQRVASKMRLAFMTPRALPLRDWELPLLASS ERITLRFGLSALRWGQGPAVLLMHGWEGRPTQFAALITALVDAGYTVVAL DGPAHGRSPGREANVVLFARAMLEAAAELPPLQAVIGHSMGGASAMLAV QLGLRTETLVSIAAPARILGVLRGFARYVGMPPRARSAFIRQVEQDVGMRA ATLDVAHYQLDMPGLIVHAEDDNFVSVKESQLIHESWFDSRLLRLEGGGH QRVLADPRVVDGVLSLLAGRSLQARQSA
	WP_108591690.1	MNTLKWVRGVNGTLGWIAPKQVASKMRTAFMTPRSLPLRDWELPLLASS ERITLRFGLSALRWGQGPAVLLMHGWEGRPTQFAALITALVEAGYTVVAL DGPAHGRSPGREANVVLFARAMLEAAAELPPLQAVIGHSMGGASAMLAV QLGLRTETLVSIAAPARILGVLRGFARYVGMPPRARSAFIRQVEQDVGMRA ATLDVAHYQLDMPGLIVHAEDDNFVSVKESQLIHESWFDSRLLRLESGGH QRVLADPRVVDGVLSLLAGRSLQARQSA
	WP_332373221.1	MTILYRGMDRTALDAAYLNTKAVPDFPALLASCQTRSAALYAATPGRRDL RYGAQPAQRFDWLPCGQPDAPLFVFIHGGYWQHCTKEDFAYAASGPLAR GYDVVLAEYTLAPVATMTDIVGEIGALLDHLAADRDGLGTAGRPIHLSGHS AGGHLTAMHRAHPAVVSALAISPLVDLEPIALCCLNDKLQLTAREVDAYSP LHHIGPGAPTVIAVGDAELPELIRQADVYATACEAAGERIARTWLRGMQHF AVLDDLATPDGAMLDALHAIAPR
	WP_034186871.1	MTILYRGMDRAALDAAYLNTKAVPDFPALLASCQARSAALYDETPGRRDL RYGAQPAQRFDWLSCGQAGAPLFVFIHGGYWQHCTKADFAYAASGPLAC GFDVILAEYTLAPVATMTGIVAEIGMLLDHLAADPDRLGTARRPIHLSGHS AGGHLTAMHRAHPAVVSALAISPLVDLEPISLCCLNDKLQLTAHEVDAYSP LRHVGPGAPTVVAVGDAELPELIRQADEYATACEAAGERIARVWLPGMQH FAVLDDLARPDGAMLAALRSITPR
	WP_130207001.1	MSALKWVRGVNGTLGWFAPKLVARKMRLAFMTPRDLPPRDWELPLLAKS ERITLRFGLSALRWGQGPAVLLMHGWEGRPTQFASLITALVDAGYTVVAL DGPAHGRSPGTEANVALFARAMLEAAAELPPLQAVIGHSMGGASAMLAV QLGLRTETLVTIAAPARILGVLRGFARYVGLPPKARSAFIRQVEKDVGMRA ATLDVAHYQLDMPGLIVHAEDDKLVSVKESQAIHEAWFDSRLLRLQEGGH QRVLADPQVIDGVLSLLAGRSLQSRQSA
	WP_1507739	MNTLKWIRGVNGTLGWIAPKRVASKMRLAFMTPRALPLRDWELPLLASSE

	89.1	RITLRFGLSALRWGQGPAVLLMHGWEGRPTQFAALITALVDAGYTVVALDGPAHGRSPGREANVVLFARAMLEAAAELPPLQAVIGHSMGGASAMLAVQLGLRTETLVSIAAPARILGVLRGFARYVGMPPRARSAFIRQVEQDVGMRAATLDVAHYQLDMPGLIVHAEDDNFVSVKESQLIHESWFDSRLLRLEGGGHQRVLADPRVVDGVLSLLAGRSLQARQSA
	WP_069745339.1	MTILYRGMDRAALDAAYLNTKAVPDFPALLASCQSRSAALYDAIAGRRELRYGALPAQRYDWLPCGQPGAPLFVFIHGGYWQHCAKEDFAYAASGPLARGYDVVLAEYTLAPTASMTDIVAEIGALLDHLAADRDGLGIAGRPIHLSGHSAGGHLTAMYRAHPAVAAALSISPLVDLEPISLCCLNDKLQLTAQEIEACSPLRHIGPGAPTVVAVGDAELPELIRQAHDYAAACDAAGERIAHVQLPGMKHFAVLDDLANPDGKMLAALRAIAPR
	WP_003226446.1	MNTLKWVRGVNGTLGWIAPQRVASKMRQAFMTPRSLPLRDWELPLLASAERITLRFGLSALRWGQGPAVLLMHGWEGRPTQFAALITALVEAGYTVVALDGPAHGRSPGREANVVLFARAMLEAAAELPPLQAVIGHSMGGASAMLAVQLGLRTETLVSIAAPARILGVLRGFARYVGMPPRARSAFIRQVEQDVGMRAATLDVAHYQLDMPGLIVHAEDDTFVSVKESQLIHESWFDSRLLRLESGGHQRVLADPRVVDGVLSLLAGRSLQARQSA
	WP_123532998.1	MNTLKWVRGVNGTLGWIAPQRVASKMRLAFMTPRVLPLRDWELPLLANSERITLRFGLSALRWGQGPTVLLMHGWEGRPTQFAALITALVDAGYTVVALDGPAHGRSPGREANVVLFARAMLEAAAELPPLQAVIGHSMGGASAMLAVQLGLRTETLVSIAAPARILGVLRGFARYVGMPRRARSEFIRQVEQDVGMRAATLDVAHYQLDMPGLIVHAEDDNFVSVKESQLIHESWFDSRLLRLEAGGHQRVLADPRVIDGVLSLLAGRSLQARQSA
	WP_102358940.1	MSTLKWVRGVNGTLGWFAPKLVASKMRLAFMTPRSLPLRDWELPLLASSERITLRFGLSALRWGQGPAVLLMHGWEGRPTQFAALITALVEAGYTVVALDGPAHGRSPGREANVVLFARAMLEAAAELPPLQAVIGHSMGGASAMLAVQLGLRTETLVSIAAPARILGVLRGFARYVGMPPRARSAFIRQVERDVGMRAATLDVAHYQLDMPGLIVHAEDDSFVSVKESQLIHESWFDSRLLRLESGGHQRVLADPRVVDGVLSLLAGRSLQARQSA
	WP_007913423.1	MNTLKWVRGVNGTLGWIAPQRVASKMRLAFMTPRSLPLRDWELPLLASSERITLRFGLSALRWGQGPTVLLMHGWEGRPTQFAALITALVDAGYTVVALDGPAHGRSPGREANVVLFARAMLEAAAELPPLQAVIGHSMGGASAMLAVQLGLRTETLVSIAAPARILGVLRGFARYVGMPPRARSAFIRQVEQDVGMRAATLDVAHYQLDMPGLIVHAEDDNFVSVKESQLIHESWFDSRLLRLEGGGHQRVLADPRVVDGVLSLLAGRSLQARQSA
	WP_150794302.1	MNTLKWVRGVNGTLGWIAPQRVASKMRLAFMTPRSLPLRDWELPLLASSERITLRFGLSALRWGQGPTVLLMHGWEGRPTQFAALITALVEAGYTVVALDGPAHGRSPGREANVVLFARAMLEAAAELPPLQAVIGHSMGGASAMLAVQLGLRTETLVSIAAPARILGVLRGFARYVGMPPRARSAFIRQVEQDVGMRAATLDVAHYQLDMPGLIVHAEDDNFVSVKESQLIHESWFDSRLLRLEGGGHQRVLADPRVVDGVLSLLAGRSLQARQSA
	WP_133210951.1	MNTLSWIRSVNGTLGRLAPEHIAGKMRHAFMTPRNLPPRDWELPLLASGERITLRFGLSALRWGQGPTVLLMHGWEGRPTQFAHLITTLVQAGYTAVALEGPAHGHSPGNQAHVALFARSLLEAAAELPPLRAVIGHSMGGASVMLALQWGLRAEMAVSVAAPAQLLGVLRNFAHRLGMPSRARAAFVRQVERDVGIPISRLDVSRYQLEIPALIAHAEDDRIVPASEALTIHQSWFDSRLLLLPEGGHQRVLSDPQLIEGVMALLLRRHSTARQSA
	WP_038994175.1	MNTLSWIRSVNGTLGHLAPEHVARKMRRAFMTPRNRPPRDWELPLLARAERITLRFGLSALRWGQGPTVLLMHGWEGRPTQFAHLIDSLVDAGYTAVALEGPAHGHSPGNEANVVLFARALLEAAAELPPLKAVVGHSMGGASMLLALQWGLRAEVAVSIAAPAQLLGVIRGFARHLGMPARARAAFIRQIERDVGVQISRLDVSGYQLELPGLIVHAEDDQLVPVDESDAIHRAWFDSRLLRLPDGGHLR

		VLADPQLREGVLALLQRSSSPARQSA
	WP_160087882.1	MNSMSWVRGFNASIGLLAPHALASKLRREFMTPHTLPPRDWELPLLAQAE<br>RITLRFGLSALRWGSGPAVLLMHGWEGRPTQFAELIKALVNAGYGVVALD<br>APAHGRSPGREANVVLFARALLEAASELPPLKAVIGHSMGGASALLATQLG<br>LRTEALVSIAAPSRILTMLRRFSHYMGLPRQARAHFVQLVEEQAGIPAGQL<br>DSAHYQLDFPGLVVHAVDDPMVPFSEAEAIHQRWFDSRLLRLERGGHQRV<br>LADPQVVQAVLTLLASLNQAPSNALAS
	NMY13981.1	MNQMAWVRGVNATLGRVAPQLIASRLRERFMTPRTTSPRDWELPLLASSE<br>RITLRFGLSALRWGSGPTVLLMHGWEGRPTQFALLIRGLVDAGYGVVALD<br>APAHGRSPGREANVVLFARALLEAASELPPLRAVIGHSMGGASALLATQM<br>GLRSETLVTIAAPSRILGLLRGFARFMGLPAEARAHFVRQVEKTAGIPAAHL<br>DVQRYRLELPGLIVHAADDQVVPVSEADLIHKAWFDSQLLRLSAGGHQRL<br>LSDPLLLQAVLELLEQVPQASLKALAS
PLA	WP_034126230.1	MGALTWVRGFNGTVGRLAPHAVASKMRRTFMTPRDLPPRDWELPLLAQS<br>ERITLRFGLSALRWGHGPAVLLMHGWEGRPTQFASLITALVDNGYSVIALD<br>GPAHGRSPGREAHVLLFARAMLEAAAELPPLYAVVGHSMGGASAMLAVQ<br>LGLRTQALVSIAAPSRFLDVLRGFTRMVGLPPRARSAFIQEVELSMGMPLK<br>HLDVAHYHLNIPGLIVHAEDDTFVPVRAAQAIHEAWFDSRLLRLEQGGHQ<br>KVLADPQVIDAVLALLAGCRLQERQSA
	WP_032896134.1	MGALTWVRGFNGTVGRLAPHAVASKMRRTFMTPRDLPPRDWELPLLAQS<br>ERITLRFGLSALRWGQGPAVLLMHGWEGRPTQFASLITALVDNGYSVIALD<br>GPAHGRSPGREAHVLLFARAMLEAAAELPPLYAVVGHSMGGASAMLAVQ<br>LGLRTQALVSIAAPSRFLDVLRGFTRMVGLPPRARSAFIQEVELSMGMPLK<br>HLDVAHYQMNIPGLIVHAEDDTFVPVRAAQAIHEAWFDSRLLRLEQGGHQ<br>KVLADPQVIDAVLALLAGCRLQERQSA
	WP_044274707.1	MGALTWVRGFNGTVGRLAPHAVASKMRRTFMTPRDLPPRDWELPLLAQS<br>ERITLRFGLSALRWGHGPAVLLMHGWEGRPTQFASLITALVDNGYSVIALD<br>GPAHGRSPGREAHVLLFARAMLEAAAELPPLYAVVGHSMGGASAMLAVQ<br>LGLRTQALVSIAAPSRFLDVLRGFTHMVGLPPRARSAFIQEVELSMGMPLK<br>HLDVAHYQMNIPGLIVHAEDDTFVPVRAAQAIHEAWFDSRLLRLEQGGHQ<br>KVLADPQVIDAVLALLAGCRLQERQSA
	ABY53108.1	MGLAIAAAAVFSLPGVATATEPTGGVQPNIVGGGNATQVYSFMVSQQSSS<br>GGHQCGGSLISSTWVVTAKHCGTPYQVRVGTTNRTSGGTVARVAQRIAHP<br>SADLALLRLSTAVPQAPVTIADASGAVGTATRIIGWGQTCAPQGGCGAPITL<br>QELNTSIVSDSRCLGISGASEICTNNPNGNSGACYGDSGGPQIKQVNGVWQ<br>LIGATSRAGNNSSTCATGPSIYVDVPYFRSWIRTNTGV
	WP_057005688.1	MGALTWIRGVNGTLGRLAPHTVANSMRRVFMTPRDLPPRDWELPLLAHA<br>ERVTLRFGLSALRWGQGPAVLLMHGWEGRPTQFASLITALVQNGYSVFAL<br>DGPAHGRSPGREAHVLLFARAMLEAAAELPPLHAVVGHSMGGASAMLAV<br>QLGLRTEALVSIAAPSRFLDVLGGFAGMVGLPSRARSAFIQEVELTLGMPL<br>KHLDVAHYQMDLPGLIVHAEDDTFVPVSASQVIHDAWFDSRLLRLEQGGH<br>QRVLADPRVVEAVLALLAGSCLQARQTA
	WP_043206304.1	MGTLTWIRRFNGTLGRLAPQTVANRMRRAFMTPRDLPPRDWELPLLAQSE<br>RITLRFGLSALRWGQGPAVLLMHGWEGRPTQFASLITALVDNGYSVIALDG<br>PAHGRSPGREAHVLLFARAMLEAAAELPPLHAVIGHSMGGASAMLAVQLG<br>LRTEALVSIASPSRFLDVLRGFTQMVGLPARARSAFIQEVELTFGMPLKHLD<br>VAHYQMNIPGLIVHAEDDTFVPVKASQAIHEAWFDSRLLRLEQGGHQKVL<br>ADPRVIDAVLALLAGRRLQALQSA
	WP_116666539.1	MGALTWVRGFNGTVGRLAPHAVASKMRRTFMTPRDLPPRDWELPLLAQS<br>ERITLRFGLSALRWGHGPAVLLMHGWEGRPTQFASLITALVGDGYSVIALD

		GPAHGRSPGREAHVLLFARAMLGAAAELPPLYAVVGHSMGGASAMLAVQ LGLRTQALVSIAAPSRFLDVLRGFTRMVGLPPRARSAFIQEVELSMGMPLK HLDVAHYQLNIPGLIVHAEDDTFVPVRAAQAIHEAWFDSRLLRLEQGGHQ KVLADPQVIDAVLALLAGCRLQERQSA
	WP_064055161.1	MMGTLTWIRRFNGTLGRLAPQTVANRMRRAFMTPRDLPPRDWELPLLAQS ERITLRFGLSALRWGQGPAVLLMHGWEGRPTQFASLITALVDNGYSVIALD GPAHGRSPGREAHVLLFARAMLEAAAELPPLHAVIGHSMGGASAMLAVQL GLRTEVLVSIASPSRFLDVLRGFTQMVGLPARARSAFIQEVELTFGMPLKHL DVAHYQMNIPGLIVHAEDDTFVPVKASQAIHEAWFDSRLLRLEQGGHQKV LADPRLIDAVLALLAGRRLQALQSA
	WP_076953514.1	MGTLTWIRRFNGTLGHLAPQTVANKMRRVFMTPRTLPPRDWELPLLAQSE RITLRFGLSALRWGQGPTVLLMHGWEGRPTQFASLITALVDKGYSVIALDG PAHGRSPGREAHVVLFARAMLEAAAELPPLRAVIGHSMGGASAMLAVQL GLRTEALVSIAAPSRCLDALRGFTTMVGLPSRARSAFIREVEMTFAMPLKH LDVAHYQMNIPGLIVHAEDDTFVPVKASQAIHEAWFDSRLLRLEQGGHQK VLADPRVIDATLSLLAGCGLQALQTA
	WP_019820855.1	MGALTWVRGFNGTVGRLAPHAVASKMRRTFMTPRDLPPRDWELPLLAQS ERITLRFGLSALRWGHGPAVLLMHGWEGRPTQFASLITALVDNGYSVIALD GPAHGRSPGREAHVLLFARAMLEAAAELPPLYAVVGHSMGGASAMLAVQ LGLRTQALVSIAAPSRFLDVLRGFTRMVGLPPRARSAFIQEVELSMGMALK HLDVAHYQLNIPGLIVHAEDDTFVPVRAAQAIHEAWFDSRLLRLEQGGHQ KVLADPQVIDAVLALLAGCRLQERQIA
	WP_042937080.1	MSTLSWIRGINGTLGRVAPRVVASRMRQMFMTPRARLPRDWELPLLATAE RITLRFGLSALRWGKGPTVLLMHGWEGRPTQFANLINALVAAGYTAVALD GPAHGRSPGREANVVVFARALLEAAAELPPLKAVVGHSMGGASAMLATQ LGLRTEALVSIAAPARVLGVLRGFARYVGLPPRARSAFIREVERDVGMRAA HLDIEHYQMDMPGLIVHAEDDRMVRVDESRRIHEAWFDSRLLRLESGGHL QVLADQRLIDGVLALLAGRSLAQRQSA
	WP_099584016.1	MGALTWIRRFNGTLGHVAPHTVANKMRRAFMTPRKLPPRDWELPLLAQS ERITLRFGLSALRWGQGPAVLLMHGWEGRPTQFASLIKALVDNGYCVIAL DGPAHGRSPGREAHVLLFARAMLEAAAELPPLHAVVGHSMGGASAMLAV QLGLRTQALVSIAAPSRFLDALRGFTRMVGLPARARSAFIQEVEMTFGMPL KYLDVAHYQMNIPGLIVHAEDDTFVPVKASQAIHDAWFDSRLLRLEQGGH QKVLADPRVIEAVLTLLAGCCLQERQSA
	WP_056785824.1	MGALTWVRGFNGTVGRLAPHAVASKMRRTFMTPRDLPPRDWELPLLAQS ERITLRFGLSALRWGHGPAVLLMHGWEGRPTQFASLITALVDNGYSVIALD GPAHGRSPGREAHVLLFARAMLEAAAELPPLYAVVGHSMGGASAMLAVQ LGLRTQALVSIAAPSRFLDVLRGFTRMVGLPPRARSAFIQEVELSMGMPLK HLDVAHYQLNIPGLIVHAEDDTFVPVRAAQAIHQAWFDSRLLRLEQGGHQ KVLADPQVIDAVLALLAGCRLQERQSA
	WP_197627415.1	MGALTWIRGFNGTVGRLAPHMVASKLRRTFMTPRNLAPRDWELPLLAQSE RITLRFGLSALRWGQGPAVLLMHGWEGRPTQFASLITALVADGYSVIALDG PAHGRSPGREAHVLLFARAMLEAAAELPPLHAVVGHSMGGASAMLAVQL GLRTEALVSIAAPSRFLDVLRGFAGMVGLPPRARSAFIHEVELTFGMPLKHL DVAHYQMNIPGLIVHAEDDTFVPVNASQAIHDAWFDSRLLRLEQGGHQKV LADPRVIDAVLSLLAGRRLQERQTA
	WP_110682084.1	MSSMSWIRGFNATVGRLAPDLVASKMHRAFLTPRDLPPRDWELPLLAESE RITLRFGLSALRWGQGPAVLLLHGWEGRPTQFAELIRALVRAGYGVVALD APTHGRSPGHEANVVLFARALLEAAGELPPLKAVIGHSLGGASALLATQLG LRTEALVTIAAPARILGALRRFAHFVGLPKQARARFVRMVEQSAGMPAAQ LDVARYQLDFPGLVVHAEDDPMVPYGEAQSIHAAWPGSRLLPLERGGHSK PLGDPRVVEAVLELLGSADLHSAVSRRVLAATVLAS

	XOQ14903.1	AQSVPWGISRVQAPAAHNRGLTGSGVKVAVLDTGISTHPDLNIRGGASFVP<br>GEPSTQDGNGHGTHVAGTIAALNNSIGVLGVAPSAELYAVKVLGASGSGS<br>VSSIAQGLEWAGNNGMHVANLSLGSPSPSATLEQAVNSATSRGVLVVAAS<br>GNSGAGSISYPARYANAMAVGATDQNNNRASFSQYGAGLDIVAPGVNVQ<br>STYPGSTYASLNGTSMATPHVAGAAALVKQKNPSWSNVQIRNHLKNTATS<br>LGSTNLYGSGLVNAEAATR
	SPU21234.1	AQSVPWGISRVQAPAAHNRGLTGSGVKVAVLDTGISTHPDLNIRGGASFVP<br>GEPSTQDGNGHGTHVAGTIAALNNSIGVLGVAPSAELYAVKVLGADGRGA<br>ISSIAQGLEWAGNNGMHVANLSLGSPSPSATLEQAVNSATSRGVLVVAASG<br>NSGASSISYPARYANAMAVGATDQNNNRASFSQYGAGLDIVAPGVNVQST<br>YPGSTYASLNGTSMATPHVAGAAALVKQKNPSWSNVQIRNHLKNTATSLG<br>STNLYGSGLVNAEAATR
	WP_0532580<br>10.1	MGALTWIRGFNGTVGRLAPRTVASKLRRTFMTPRNLPPRDWELPLLAQSE<br>RITLRFGLSALRWGQGPAVLLMHGWEGRPTQFASLITALVDNGYSVIALDG<br>PAHGRSPGREAHVLLFARAMLEAAAELPPLQAVVGHSMGGASALLAVQL<br>GLRTEALVSIAAPSRFLDVLRGFAGMVGLPARARAAFIREVELTFGMPLKH<br>LDVAHYQMNLPGLIVHAEDDTFVPVKASQAIHDAWFDSRLLRLEQGGHQK<br>VLADPRVIDGVLTLLAGCRLQARQTA
	WP_0995254<br>09.1	MNQMTWVRGVNATLGRVAPQLIASRLRERFMTPRTQPPRDWELPLLASAE<br>RITLRFGLSALRWGSGPTVLLMHGWEGRPTQFALLIRGLVDAGYGVIALDA<br>PAHGRSPGREANVVLFARALLEAASELPPLRAVIGHSMGGASALLATQMG<br>LRCETLVTVAAPSRILGLLRGFARFMGLPAEARAHFVRAVETTAGIPAAHL<br>DVQRYQLDLPGLIVHAEDDQVVPVGEADLIHKAWFDSQLLRLPAGGHQRL<br>LSDPLLLQAVLELLEQVPQASLKALAS
	WP_0540638<br>32.1	MNTLRWIRGINGTLGRVAPRVAASRMRQVFMTPRERSPRDWELPLLATAE<br>RITLRFGLSALRWGQGPTVLLMHGWEGRPTQFASLIEALVAAGYTAVALD<br>GPAHGQSPGHEANVVAFARALLEAAAELPPLKAVIGHSMGGASAMLATQL<br>GLRTEALVSIAAPARVLGVLRGFAQHVGLPPRARSAFIREVERDVGMRAEH<br>LDIGHYQMDMPGLIVHAEDDQLVAVDESRRIHEAWFDSRLLRLESGGHQR<br>VLADPRLIDGVLALLAGRSMAQRQSA
	WP_0714881<br>09.1	MGTLTWVRRFNSTLGHLVPQTVANRMRRAFMTPRELPPRDWELPLLAQSE<br>RITLRFGLSALRWGQGPAVLLMHGWEGRPTQFASLISALVDNGYSVIALDG<br>PAHGRSPGREAHVLLFARAMLEAAAELPPLHAVIGHSMGGASAMLAVQLG<br>LRTEALVSIAAPSRFLDVLRGFTRMVGLPARARSAFIQEVELTFGMPLKHLD<br>VAHYQMNIPGLIVHAEDDTFVPVKASQAIHEAWFDSRLLRLEQGGHQKVL<br>ADPRVIDAVLALLAGCRLQERQSA
	WP_0714863<br>26.1	MGALTWIRGFNGTVGRLAPHTVASKMRRTFMTPRDLPPRDWELPLLAQSE<br>RITLRFGLSALRWGQGPAVLLMHGWEGRPTQFASLITALVDNGYSVIALDG<br>PAHGRSPGREAHVLLFARAMLEAAAELPPLHAVVGHSMGGASAMLAVQL<br>GLRTQALVSIAAPSRFLDVLRGFAGMVGLPPRARSAFIQEVELTFGMPLKH<br>LDVAHYQMNIPGLIVHAEDDTFVPVKASQQIHDTWFDSRLLRLEQGGHQK<br>VLADPRVIDAVLALLAGRRLQERQTA
	WP_1258815<br>20.1	MNQMTWVRGVNATLGRVAPQLIASRLRERFMTPRTQPPRDWELPLLASAE<br>RITLRFGLSALRWGSGPTVLLMHGWEGRPTQFALLIRGLVDAGYGVIALDA<br>PAHGRSPGREANVVLFARALLEAASELPPLRAVIGHSMGGASALLATQMG<br>LRCETLVTVAAPSRILGLLRGFARFMGLPAEARAHFVRAVETTAGIPAAHL<br>DVQRYQLDLPGLIVHAEDDQVVPVGEADLIHKAWFDSQLLRLPAGGHQRL<br>LSDPLLLQAVLELLEQVPQASLKAMAS
	WP_0549216<br>54.1	MGALTWVRGFNGTVGRLAPHAVASKMRRTFMTPRDLPPRDWELPLLAQS<br>ERITLRFGLSALRWGHGPAVLLMHGWEGRPTQFASLITALVDNGYSVIALD<br>GPAHGRSPGREAHVLLFARAMLEAAAELPPLYAVVGHSMGGASAMLAVQ<br>LGLRTQALVSIAAPSRFLDVLRGFTRMVGLPPRARSAFIQEVELSMGMPLK

		HLDVAHYQLNIPGLIVHAEDDTFVPVRAAQAIHEAWFDSRLLRLEQGGHQKVLADPQVIDAVLALLAGCRLQERQSA
	WP_169851737.1	MSTLSWIRRVNGTVGRLAPQTIANQMRRAFMTPRDLPPRDWELPLLAQAERVTLRFGLSALRWGQGPAVLMMHGWEGRPTQFASLIDALVGAGYSVIALDGPAHGRSPGREAHVVLFARAMLEAAAELPPLHAVVGHSMGGASAMLAIQLGLRTNALVSIAAPSRLLDVLRGFAGVVGMPARARAAFIQEVEYSLGIPLKHLDVAHYQMNIPGLIVHAEDDTFVPVKASQMIHEAWFDSRLLRLEQGGHQKVLADPRVVEGVLALLAGCREPTRQTA
	WP_058426895.1	MGTLTWIRRFNGTLGHLAPQTVANRMRRAFMTPRDLPLRDWELPLLAQSERITLRFGLSALRWGQGPAVLLMHGWEGRPTQFASLISALVDNGYSVIALDGPAHGRSPGREAHVLLFARAMLEAAAELPPLHAVIGHSMGGASAMLAVQLGLRTEALVSIAAPSRFLDVLRGFTKMVGLPARARSAFIQEVELTFGMPLKHLDVAHYQMNIPGLIVHAEDDTFVPVKASQAIHEAWFDSRLLRLEQGGHQKVLADPRVIDGVLALLAGARLQERQTA
	WP_003192569.1	MGALTWVRGFNGTVGRLAPHAVASKMRRTFMTPRDLPPRDWELPLLAQSERITLRFGLSALRWGHGPAVLLMHGWEGRPTQFASLITALVDNGYSVIALDGPAHGRSPGREAHVLLFARAMLEAAAELPPLYAVVGHSMGGASAMLAVQLGLRTQALVSIAAPSRFLDVLRGFTRMVGLPPRARSAFIQEVELSMGMPLKHLDVAHYQMNIPGLIVHAEDDTFVPVRAAQAIHEAWFDSRLLRLEQGGHQKVLADPQVIDAVLALLAGCRLQERQSA
	WP_060765121.1	MGALTWVRGFNGTVGRLAPHAVASKMRRTFMTPRDLPPRDWELPLLAQSERITLRFGLSALRWGHGPAVLLMHGWEGRPTQFASLIAALVDNGYSVIALDGPAHGRSPGREAHVLLFARAMLEAAAELPPLYAVVGHSMGGASAMLAVQLGLRTQALVSIAAPSRFLDVLRGFTRMVGLPPRARSAFIQEVELSMGMPLKHLDVAHYQLNIPGLIVHAEDDTFVPVRAAQAIHEAWFDSRLLRLEQGGHQKVLADPQVIDAVLALLAGCRLQERQSA
	WP_154743290.1	MSTFKWTRGVNGALGRLAPQIIASKMRRVFMTPRNFPPRDWELPLLAQSERITLRFGLSALRWGQGPTVLLMHGWEGRPTQFASLISALVGAGYSVIALEGPAHGRSPGREAHVLLFARAMLEAAAELPPLHAVIGHSMGGASAMLAVQLGLRTEALVSIAAPSRFLDVLRGFAGMVGLPARARSAFIQEVEHAFGMPLKYLDVAHYQMNMPGLIVHAEDDTFVSVRASQVIHEAWFDSRLMRLKQGGHQKVLADPHVIKGVLALLAGCRPAQRQTA
